# Lineage-specific mutation of *Lmx1b* provides new insights into distinct regulation of suture development in different areas of the calvaria

**DOI:** 10.3389/fphys.2023.1225118

**Published:** 2023-08-01

**Authors:** Angel Cabrera Pereira, Krishnakali Dasgupta, Thach-Vu Ho, Maria Pacheco-Vergara, Julie Kim, Niam Kataria, Yaowei Liang, Jeslyn Mei, Jinyeong Yu, Lukasz Witek, Yang Chai, Juhee Jeong

**Affiliations:** ^1^ Department of Molecular Pathobiology, College of Dentistry, New York University, New York, NY, United States; ^2^ Center for Craniofacial Molecular Biology, University of Southern California, Los Angeles, CA, United States; ^3^ Department of Psychology, Hunter College, City University of New York, New York, NY, United States; ^4^ Department of Biology, College of Arts and Sciences, New York University, New York, NY, United States; ^5^ Biomaterials Division, New York University College of Dentistry, New York, NY, United States; ^6^ Hansjörg Wyss Department of Plastic Surgery, New York University Grossman School of Medicine, New York, NY, United States; ^7^ Department of Biomedical Engineering, New York University Tandon School of Engineering, Brooklyn, NY, United States

**Keywords:** calvaria, craniosynostosis, LMX1B, craniofacial development, mouse, embryo

## Abstract

The calvaria (top part of the skull) is made of pieces of bone as well as multiple soft tissue joints called sutures. The latter is crucial to the growth and morphogenesis of the skull, and thus a loss of calvarial sutures can lead to severe congenital defects in humans. During embryogenesis, the calvaria develops from the cranial mesenchyme covering the brain, which contains cells originating from the neural crest and the mesoderm. While the mechanism that patterns the cranial mesenchyme into bone and sutures is not well understood, function of *Lmx1b*, a gene encoding a LIM-domain homeodomain transcription factor, plays a key role in this process. In the current study, we investigated a difference in the function of *Lmx1b* in different parts of the calvaria using neural crest-specific and mesoderm-specific *Lmx1b* mutants. We found that *Lmx1b* was obligatory for development of the interfrontal suture and the anterior fontanel along the dorsal midline of the skull, but not for the posterior fontanel over the midbrain. Also, *Lmx1b* mutation in the neural crest-derived mesenchyme, but not the mesoderm-derived mesenchyme, had a non-cell autonomous effect on coronal suture development. Furthermore, overexpression of *Lmx1b* in the neural crest lineage had different effects on the position of the coronal suture on the apical part and the basal part. Other unexpected phenotypes of *Lmx1b* mutants led to an additional finding that the coronal suture and the sagittal suture are of dual embryonic origin. Together, our data reveal a remarkable level of regional specificity in regulation of calvarial development.

## 1 Introduction

The calvaria (top part of the skull) comprises bone plates and soft tissue joints, called sutures. During fetal and early postnatal stages, large areas of soft tissue appear where multiple sutures intersect, and they are called fontanels. There are five pieces of bone in the mammalian calvaria. In mice, they are a pair of the frontal bone, a pair of the parietal bone, and a single piece of the interparietal bone. The interfrontal suture (also known as the metopic suture) joins the two frontal bones, and the sagittal suture connects the two parietal bones. In addition, the coronal suture lies between the frontal bone and the parietal bone, whereas the lambdoidal suture connects the parietal bone and the interparietal bone. While the bone provides physical protection to the brain, the sutures contain progenitors and stem cells for osteoblasts, which allow continued growth of the skull to accommodate the rapidly expanding brain during childhood (Ishii et al., 2015; Twigg and Wilkie, 2015; White et al., 2021; Stanton et al., 2022). Craniosynostosis (premature fusion of one or more sutures) is a major type of birth defects in humans, found in 1 out of ∼2300 live births (Boulet et al., 2008; Levi et al., 2012; Stanton et al., 2022). It can profoundly affect overall morphology of the skull, and even impair development of the brain through an increase in intracranial pressure (Levi et al., 2012; Yu et al., 2021; Stanton et al., 2022). Therefore, investigating how the sutures are generated and maintained is highly relevant to human health.

During embryogenesis, the calvaria develops from the head mesenchyme layer covering the brain (cranial mesenchyme), which also gives rise to the meninges and the dermis of the scalp (Ishii et al., 2015; Ferguson and Atit, 2019). This mesenchyme contains cells from the neural crest and the mesoderm. Lineage-tracing studies in mice have found that the frontal bone, the interfrontal suture, and the sagittal suture are made of the neural crest-derived cells, whereas the parietal bone and the coronal suture are made of the mesoderm-derived cells (Jiang et al., 2002; Yoshida et al., 2008). The interparietal bone has cells from both origins. A more recent report has shown that the frontal bone receives a minor contribution of mesoderm-derived cells also (Deckelbaum et al., 2012).

The calvarial bone develops through intramembranous ossification, and this process begins around embryonic day (E) 12 in mice, with expression of an osteogenic gene *Runx2* in the condensed mesenchyme just above the eye. This region is called the supra-orbital mesenchyme (Deckelbaum et al., 2012; Ferguson and Atit, 2019; Galea et al., 2021), and it contains rudiments of the frontal bone and the parietal bone separated by presumptive coronal suture progenitors. Between E12 and birth [= E19 or postnatal day (P) 0], the frontal bone, the coronal suture, and the parietal bone expand apically side-by-side. The interfrontal suture and the sagittal suture are established when the osteogenic fronts from the left and the right approximate at the dorsal midline late in gestation (Yoshida et al., 2008; Deckelbaum et al., 2012; Ishii et al., 2015).

The calvarial bone and sutures are organized in a stereotypic pattern in each species, but the mechanism that determines the layout remains poorly understood (Teng et al., 2019; White et al., 2021). It is thought to involve both genetic regulations and mechanical forces (Galea et al., 2021). For example, a computational model that combined the mechanical strain from the brain growth with the Turing reaction-diffusion model was able to accurately predict positions of the bone and sutures in the normal calvaria (Lee et al., 2019). Furthermore, we have shown that a large number of genes are differentially expressed along the apical-basal axis of the cranial mesenchyme, and one such gene, *Lmx1b* (LIM homeobox transcription factor 1 beta), plays a crucial role in early calvarial patterning (Cesario et al., 2018; Dasgupta et al., 2019).

LMX1B is a key transcriptional regulator for development of multiple body parts including the limb, the brain, the kidney, and the calvaria (Chen et al., 1998a; Chen et al., 1998b; Morello et al., 2001; Guo et al., 2007). In humans, *LMX1B* mutation underlies the Nail-Patella syndrome, affecting the nails, kneecaps, and the kidney, and it was also detected in one case of familial craniosynostosis (Chen et al., 1998a; Morello et al., 2001; Wilkie et al., 2010). In the cranial mesenchyme, *Lmx1b* is specifically expressed in the area apical to the supra-orbital mesenchyme (also known as early-migrating mesenchyme) and inhibits osteogenesis in this region. Deletion of *Lmx1b* using *Prrx1-Cre* (Logan et al., 2002), which is broadly active in the cranial mesenchyme, led to heterotopic ossification at the vertex and fusion of multiple sutures (Cesario et al., 2018).

In the current study, we have further dissected *Lmx1b* function in the developing calvaria through lineage-specific deletion and overexpression. We defined the extent of the requirement of *Lmx1b* in the calvaria. In addition, there was a difference in *Lmx1b* function from the neural crest-side and the mesoderm-side of the coronal suture. Lastly, by analyzing the coronal suture at multiple points along the apical-basal axis, we obtained results suggesting distinct regulation at different positions.

## 2 Materials and methods

### 2.1 Animals

The mouse lines used in this study have been described previously: *Sox10-Cre* [=Tg(Sox10-cre)1Wdr (Matsuoka et al., 2005)], *Mesp1-Cre* [=Mesp1^tm2(cre)Ysa^ (Saga et al., 1999)], *Lmx1b* ^*fl*^ [=Lmx1b^tm4.1Rjo^ (Suleiman et al., 2007)], *R26* ^*Lmx1b*^ [=Gt(ROSA)26Sor^tm1(CAG-Lmx1b,ALPP)Rjo^ (Li et al., 2010)], *R26* ^*R-YFP*^ [=Gt(ROSA)26Sor^tm1(EYFP)Cos^ (Srinivas et al., 2001)]. The mice were maintained in C57BL/6J background.

Because *Sox10-Cre* is active in the male germ line (Crispino et al., 2011), *Sox10-Cre;Lmx1b* ^*fl/fl*^ mutants were obtained from a cross of female *Sox10-Cre;Lmx1b* ^*fl/+*^ *x* male *Lmx1b* ^*fl/fl*^, and *Sox10-Cre;Lmx1b* ^*fl/fl*^;*R26* ^*R-YFP/+*^ mutants from female *Sox10-Cre;Lmx1b* ^*fl/+*^ *x* male *Lmx1b* ^*fl/+*^;*R26* ^*R-YFP/R-YFP*^. *Mesp1-Cre;Lmx1b* ^*fl/fl*^ mutants were obtained from a cross of male *Mesp1-Cre;Lmx1b* ^*fl/+*^ × female *Lmx1b* ^*fl/fl*^, and *Mesp1-Cre;Lmx1b* ^*fl/fl*^;*R26* ^*R-YFP/+*^ mutants from male *Mesp1-Cre;Lmx1b* ^*fl/+*^ × female *Lmx1b* ^*fl/+*^;*R26* ^*R-YFP/R-YFP*^. *Sox10-Cre;R26* ^*Lmx1b/+*^ mutants were obtained from a cross of female *Sox10-Cre* x male *R26* ^*Lmx1b/+*^, and *Sox10-Cre;R26* ^*R-YFP/Lmx1b*^ mutants from female *Sox10-Cre;R26* ^*R-YFP/+*^ × male *R26* ^*Lmx1b/+*^ or *R26* ^*Lmx1b/Lmx1b*^. *Sox10-Cre;R26* ^*R-YFP/+*^ and *Mesp1-Cre;R26* ^*R-YFP/+*^ samples were obtained from the above crosses, and some were also from female *Sox10-Cre;R26* ^*R-YFP/+*^ × male wild type C57BL/6J, and male *Mesp1-Cre* x female *R26* ^*R-YFP/R-YFP*^.

The sample size for each experiment is indicated in the figure legends and the text of the Results section, and the phenotypes were consistent in all samples unless indicated otherwise. We did not separate the samples by sex because we found comparable calvarial phenotypes in male and female *Lmx1b* mutants in a previous study using *Prrx1-Cre* (Cesario et al., 2018). All the animal work was performed following a protocol approved by Institutional Animal Care and Use Committee of New York University.

### 2.2 Microcomputed tomography (microCT), skeletal staining, alkaline phosphatase staining

**FIGURE 1:**
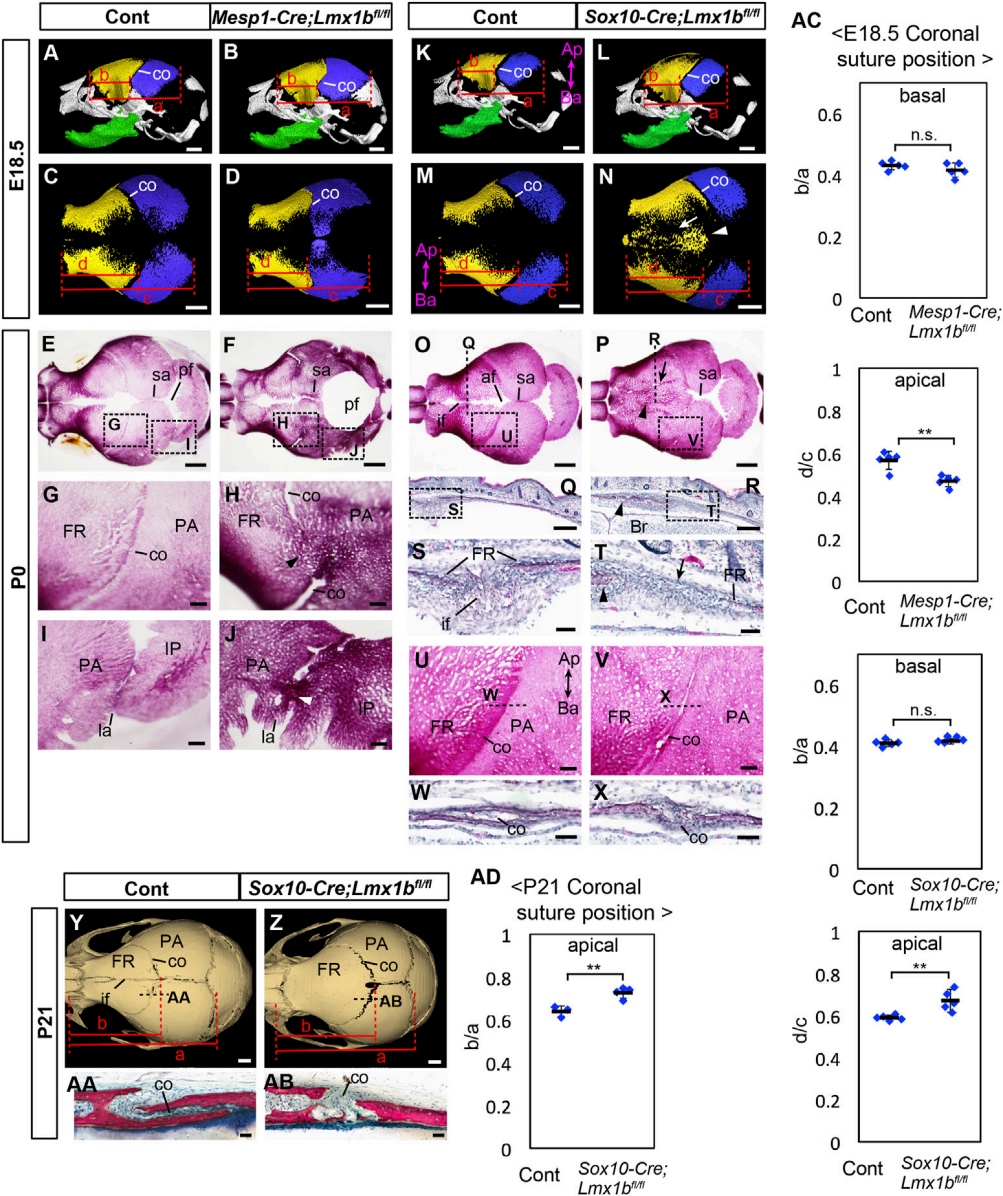
Calvarial phenotypes of mesoderm-specific and neural crest-specific *Lmx1b* deletion mutants. **(A–D, K–N)** 3D reconstruction from microCT scans of E18.5 skulls. Yellow: frontal bone (FR), blue: parietal bone (PA), green: dentary bone. **(A, B, K, L)** are lateral views of the whole skull. **(C, D, M, N)** are superior views of the frontal bone and the parietal bone only. 5 samples per genotype were examined. The arrow and the arrowhead in N point to a suture-like gap off the midline and the heterotopic bone at the midline, respectively. **(E–J, O, P, U, V)** Superior views of P0 skulls stained with Alizarin red for bone. **(G–J, U, V)** show the boxed areas in **(E, F, O, P)**. **(Q–T, W, X)** Coronal sections **(Q–T)** and sagittal sections **(W, X)** of P0 heads stained with hematoxylin and eosin. Approximate positions of the sections are indicated by dotted lines in (**O, P, U, V, S, T)** show the boxed areas in **(Q, R)**, respectively. The arrowheads in **(H, J)** point to the fusion of the coronal suture and the lambdoidal suture, respectively. The arrows and the arrowheads in **(P, R, T)** point to a suture-like gap off the midline and the heterotopic bone at the midline, respectively. For **(E–J)**, 7 controls and 6 mutants were examined. For **(O, P, U, V)** 12 controls and 7 mutants were examined. For **(Q–T, W, X)** 2 controls and 3 mutants were examined. For **(W, X)** 2 controls and 2 mutants were examined. **(Y, Z)** Superior views of 3D reconstruction from microCT scans of P21 skulls. 3 controls and 4 mutants were examined. **(AA, AB)** Sagittal sections of P21 heads stained with Stevenel׳s Blue and Van Gieson׳s Picro Fuschin. Approximate positions of the sections are indicated by dotted lines in **(Y, Z)**. **(AC)** Relative position of the coronal suture (co) at E18.5 measured as a ratio of b to a in **(A, B, K, L)** (basal) or d to c in **(C, D, M, N)** (apical). The dotted lines through selected landmarks were drawn perpendicular to the anterior-posterior axis of the head, and the distance between the two dotted lines were measured. The landmarks for each measurement are a: from the anterior end of the frontal bone to the posterior end of the parietal bone, b: from the anterior end of the frontal bone to the coronal suture next to the anterior end of the parietal bone, c: from the anterior end of the frontal bone to the posterior end of the parietal bone, d: from the anterior end of the frontal bone to the posterior end of the frontal bone facing the parietal bone. **(AD)** Relative position of the coronal suture at P21 measured as a ratio of b to a in **(Y, Z)**. The landmarks are a: from the anterior end of the frontal bone to the posterior end of the parietal bone, b: from the anterior end of the frontal bone to the posterior end of the frontal bone. All the controls in this figure are *Lmx1b* ^*fl/fl*^ or *Lmx1b* ^*fl/+*^. Bar in **(A–F, K–P, Y, Z)**: 1 mm, bar in **(G–J, Q, R, U–V)**: 0.2 mm, bar in **(S, T, W, X, AA, AB)**: 0.05 mm af: anterior fontanel, Ap-Ba, apical-basal axis of the calvaria; co, coronal suture; FR, frontal bone; if, interfrontal suture; IP, interparietal bone; la, lambdoidal suture; PA, parietal bone; pf, posterior fontanel; sa, sagittal suture. **: *p* < 0.01, n.s.: not statistically significant (*p* > 0.05).

MicroCT of E18.5 skulls was performed with Scanco V1.2a at 10 μm resolution. Volume rendering was done with Amira (Figure 1) or Avizo (Figure 5) (Thermo Fisher Scientific). MicroCT of P21 skulls was performed with Skyscan 1172 at 8.5 μm resolution, and volume rendering was done with 3D slicer (Fedorov et al., 2012).

For Alizarin Red staining of the skulls, the heads were skinned and incubated in ethanol for 2 days and acetone for 2 days and stained with 0.01% Alizarin Red S in 1% KOH solution for 2 days. The samples were cleared in 1% KOH and stored in glycerol. For alkaline phosphatase staining, the surface ectoderm was removed, and the heads were stained with NBT/BCIP (Sigma B6404).

### 2.3 Preparation of frozen sections, immunofluorescence, and RNA *in situ* hybridization

Frozen sections were prepared at 12 μm thickness as previously described (Jeong et al., 2012). For transverse sections, a section’s position along the apical-basal axis was calculated as (No. of sections between the top of the head and the section of interest) divided by (No. of sections between the top of the head and the top of the eye), rounded to one digit below the decimal point. We tallied all sections including those lost in the process. “Top of the head” in this context was defined as the area of the head that would appear on the first section when we cut through the head embedded upside down. Its precise location varied slightly from one sample to another because different heads were tilted slightly differently during embedding. Generally speaking, the top of the head was at or close to the dorsal midline in terms of the medio-lateral position, and around the posterior end of the cerebral hemispheres in terms of the antero-posterior position, because when this area was touching the bottom of the embedding mold, the inverted head tended to stand still.

Immunofluorescence was performed on frozen sections as previously described (Jeong and McMahon, 2005). The primary antibodies were chicken anti-green fluorescence protein (Abcam, ab13970) and rabbit anti-SP7 (Abcam, ab209484). Alexa Fluorophore-conjugated secondary antibodies (Thermo Fisher Scientific) were used for detection. DAPI (40,6-diamidino-2 phenylindole) was used to stain the nuclei.

RNA *in situ* hybridization for *Lmx1b* was performed on frozen sections using RNAscope Multiplex Fluorescent Reagent Kit v2 (Advanced Cell Diagnostics, 323100) and an RNAscope probe for *Lmx1b* (Advanced Cell Diagnostics, 412931) according to the manufacturer’s protocol. It was followed by immunofluorescence for SP7, for which the secondary antibody was donkey anti-rabbit IgG, horseradish peroxidase-conjugated (Novex, A16023), with Cy3-tyramide (PerkinElmer, NEL74001KT) as a substrate.

Fluorescence pictures showing specific areas of the tissue were captured with Nikon Eclipse E600, except for *Lmx1b* fluorescence RNA *in situ* hybridization images, which were acquired with Leica SP8 confocal microscope. Whole-section pictures were captured with Nikon SMZ1500 in tiles and manually stitched with Adobe Photoshop.

### 2.4 Histological staining of sections

P21 heads were fixed in 4% paraformaldehyde solution, dehydrated through an ethanol series, and embedded in methyl methacrylate. The samples were sectioned into 400 µm-thick slices with a precision diamond saw (Isomet 2000, Buehler Ltd). The sections were glued to an acrylic plate with a photolabile acrylate-based adhesive (Technovit 7210 VLC adhesive, Heraeus Kulzer GMBH) before grinding and polishing to a final thickness of ∼200 µm. The sections were subsequently stained with Stevenel׳s Blue and Van Gieson׳s Picro Fuschin, and imaged with a slide scanner (Aperio Technologies).

For histology of P0 heads, frozen sections from Section 2.3 were stained with hematoxylin and eosin, and imaged with a slide scanner (Aperio Technologies).

### 2.5 Quantitative analysis of images and statistical tests

Volume measurements from microCT data were obtained by Amira (Supplementary Figure S2) or Avizo (Figure 5) (Thermo Fisher Scientific). The length and area measurements were done by FIJI (Schindelin et al., 2012) from images acquired at consistent settings between controls and mutants. Allometry was not considered. A comparison between genotypes was made with samples at the same developmental stage. We did not employ additional standardization across samples. However, except for the bone volume, our data were represented as a ratio of two measurements from the same sample, which reduced the effect of variation in the overall size of the head. All samples were measured without their genotypes indicated. Two-tailed Student’s t-test was used for a comparison between control and mutant samples, and *p* < 0.05 was considered significant. In the charts, values from individual samples are presented with an average (bar) and standard deviation as error bars. For incidental data, Fisher’s exact test was used to calculate *p* values.

## 3 Results

Our previous study (Cesario et al., 2018) had left a couple of important questions unanswered regarding the function of *Lmx1b*. One of them was whether *Lmx1b* is required for development of all sutures in the calvaria. In *Prrx1-Cre;Lmx1b* ^*fl/-*^ mutants, the aberrant ossification excluded the anterior half of the interfrontal suture, the basal part of the coronal suture, the sagittal suture, and the posterior fontanel. We had noted that the basal coronal suture did not express *Lmx1b,* and thus we concluded that this region developed independently of *Lmx1b* function (Cesario et al., 2018). However, it was unclear why other areas remained unossified in *Prrx1-Cre;Lmx1b* ^*fl/-*^ mutants. One possibility was that these regions were occupied by cells still expressing *Lmx1b* in the mutants, since *Prrx1-Cre* was inactive in some parts of the cranial mesenchyme (Logan et al., 2002; Cesario et al., 2018). To answer this question, we used *Mesp1-Cre* to delete *Lmx1b* in the mesoderm-derived cells, and *Sox10-Cre* to delete *Lmx1b* in the neural crest-derived cells, as the two lineages make up the whole calvaria (Saga et al., 1999; Jiang et al., 2002; Matsuoka et al., 2005; Yoshida et al., 2008). Furthermore, this approach allowed us to address another important question, i.e., whether *Lmx1b* has any non-cell autonomous function in inhibiting osteogenesis, by examining the phenotype at the lineage boundary. Lastly, although we have shown that LMX1B does not inhibit osteogenesis in the limbs (Cesario et al., 2018), which undergoes endochondral ossification, it was unknown whether LMX1B has an anti-osteogenic function in the facial mesenchyme, which undergoes intramembranous ossification just like the calvaria (Galea et al., 2021). *Lmx1b* expression was evident in the nose and the mandible from E13.5 (Schweizer et al., 2004) (Supplementary Figure S2). *Prrx1-Cre* has limited activity in these areas (Logan et al., 2002), but *Sox10-Cre* can be effective because the nose and the mandible contain mostly neural crest-derived mesenchyme.

We did not use *Wnt1-Cre* for neural crest-specific mutation because it is active in a significant part of the brain in addition to the neural crest (Danielian et al., 1998; Keuls and Parchem, 2021). The *Wnt1-Cre* domain in the brain extensively overlaps with *Lmx1b* expression along the dorsal midline and in the midbrain (Guo et al., 2007; Mishima et al., 2009). Because *Lmx1b* plays an important role in brain development (Guo et al., 2007), *Wnt1-Cre*-mediated mutation of *Lmx1b* can lead to calvarial defects that are secondary to the brain defects.

### 3.1 Mesoderm-specific deletion of *Lmx1b* results in partial fusion of the coronal suture and the lambdoidal suture but no ossification in the posterior fontanel

We used microCT and Alizarin Red staining to examine the morphology of the calvaria at E18.5 and P0 (Figure 1). In E18.5 *Mesp1-Cre;Lmx1b* ^*fl/fl*^ mutants (*n* = 5), the apical end, but not the basal end, of the coronal suture was displaced anteriorly within the frontal bone-coronal suture-parietal bone unit (Figures 1A–D, AC; see Supplementary Figure S1 for definitions of different axes). The parietal bone was enlarged while the frontal bone was reduced (Supplementary Figures S2A, B, G). The morphology of the coronal suture exhibited a complex phenotype. There was partial fusion of the coronal suture affecting all mutants examined at P0 (*n* = 6, *p* = 0.00058), either unilaterally (2 out of 6 mutants) or bilaterally (4 out of 6 mutants). The fusion was always only in the middle part along the apical-basal axis (Figures 1E–H). Furthermore, the apical part and the basal part were completely disjointed, with the apical part located significantly anterior to the expected position based on the trajectory from the basal part (Figure 1H).

The lambdoidal suture was also affected in all mutants examined (*n* = 6, *p* = 0.00058), with partial fusion either unilaterally (2 out of 6 mutants) or bilaterally (4 out 6 mutants), and it had irregular morphology even where unfused (Figures 1I, J). However, the posterior fontanel remained unossified and became a large soft spot in the skull with the bulging brain, similar to what was described in *Prrx1-Cre;Lmx1b* ^*fl/-*^ mutants (Figure 1F) (Cesario et al., 2018). We could not examine *Mesp1-Cre;Lmx1b* ^*fl/fl*^ mutants at postnatal stages because the newborns were visibly in distress with brain hemorrhage and thus had to be euthanized.

### 3.2 Neural crest-specific deletion of *Lmx1b* results in ossification of the entire interfrontal suture and abnormal morphology of the coronal suture, but the sagittal suture remains patent

In E18.5 and P0 *Sox10-Cre;Lmx1b* ^*fl/fl*^ mutants (*n* = 12), the entire interfrontal suture and the anterior fontanel were occupied by heterotopic bone (arrowheads in Figures 1N, P, R, T), while the sagittal suture appeared widened (Figure 1O–P). Also, there was a suture-like gap off the midline between the heterotopic bone and the endogenous frontal bone (arrows in Figure 1N, P, T). The apical end, but not the basal end, of the coronal suture was displaced posteriorly within the frontal bone-coronal suture-parietal bone unit (Figures 1K–N, AC). These changes were concomitant with an increase in frontal bone volume (Supplementary Figures S2C, D, G). Although the coronal suture was patent in *Sox10-Cre;Lmx1b* ^*fl/fl*^ mutants, the morphology was affected in all samples examined at P0 (*n* = 7, *p* = 0.00002). The characteristic overlap between the parietal bone and the frontal bone was lost, unilaterally (2 out of 7 mutants) or bilaterally (5 out of 7 mutants) (Figures 1U, V). This phenotype was confirmed on sections (Figures 1W, X). At P21 (*n* = 3), the coronal suture showed the same phenotype as P0, i.e., posterior displacement at the apical end and a loss of bone overlap (Figures 1Y, Z, AA, AB, AD), but the frontal bone had become a continuous piece without any gap (Figure 1Y, Z). We also measured the volumes of the nasal bone and the dentary bone in *Sox10-Cre;Lmx1b* ^*fl/fl*^ mutants at P0, but did not find a significant difference from controls (Supplementary Figures S2C–F, G).

### 3.3 The sagittal suture and the coronal suture are of dual embryonic origin, whereas the lambdoidal suture develops from the mesoderm

Because the sagittal suture had been known to be neural crest-derived (Jiang et al., 2002; Yoshida et al., 2008), it was unexpected that the sagittal suture was patent in *Sox10-Cre;Lmx1b* ^*fl/fl*^ mutants (Figure 1). Therefore, we scrutinized the embryonic origin in detail using a yellow fluorescent protein (YFP) Cre reporter (Srinivas et al., 2001) and a preosteoblast marker SP7 (Supplementary Figure S3). We discovered that, in normal embryos, only the anterior half of the sagittal suture mesenchyme was neural crest-derived whereas the posterior half was mesoderm-derived (Supplementary Figures S3H, I, N, O). We also examined the embryonic origin of the sagittal suture in *Lmx1b* mutants. In *Mesp1-Cre;Lmx1b* ^*fl/fl*^ *;R26* ^*R-YFP/+*^ mutants (*n* = 3), the composition of the sagittal suture mesenchyme resembled that of the controls (Supplementary Figures S3K, L). In other words, the mesodermal cells were able to contribute to the posterior sagittal suture mesenchyme even in the absence of *Lmx1b*. In contrast, in *Sox10-Cre;Lmx1b* ^*fl/fl*^ mutants (*n* = 3), the whole sagittal suture was made of cells from the mesoderm, in which *Lmx1b* was not deleted (Supplementary Figure S3R). A section through a position that would normally be the anterior sagittal suture showed that the neural crest-derived cells became bone (arrowhead in Supplementary Figure S3Q), while the mesoderm-derived cells formed the suture mesenchyme next to the parietal bone (open arrowheads in Supplementary Figure S3Q).

**FIGURE 2:**
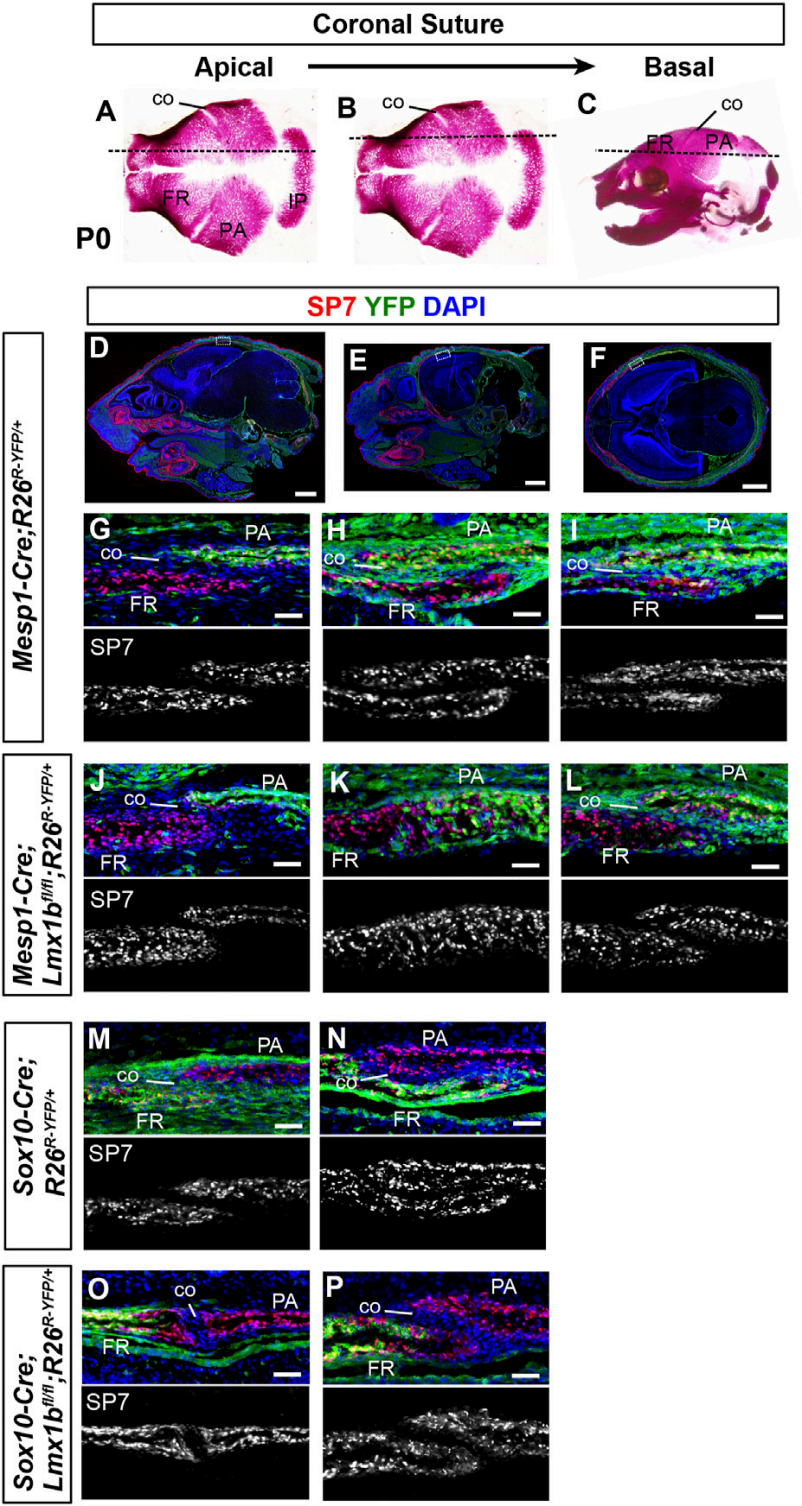
Contribution of mesoderm-derived cells and neural crest-derived to the coronal suture **(A–C)** Normal skulls at P0 stained with Alizarin red for bone. The dotted lines indicate an approximate plane of section for all the panels in each column, starting from the apical part of the coronal suture **(A)** and progressing in a basal direction. **(D–F)** Sagittal sections **(D, E)** and a transverse section **(F)** of P0 *Mesp1-Cre;R26* ^*R-YFP/+*^ heads processed by immunofluorescence for SP7 and YFP. Whole-section images are presented to help readers assess the plane of section. The boxes in **(D–F)** mark the tissue regions shown in **(G–I)**, respectively, and the panels below in the same column **(J–P)** are from equivalent regions of the animals of the indicated genotype. 3 samples per genotype were examined for each plane of section. Bar in **(D–F)**: 1 mm, bar in **(G–P)**: 0.05 mm.

As to the coronal suture, it was fused along the entire length in *Prrx1-Cre;Lmx1b* ^*fl/-*^ mutants except for the basal part, which indicated that *Lmx1b* was required in the apical and the middle parts (Cesario et al., 2018). Based on the notion that the coronal suture is mesoderm-derived, we were unable to explain why the apical part was not fused in *Mesp1-Cre;Lmx1b* ^*fl/fl*^ mutants (Figure 1). Similarly, it was unclear why the coronal suture morphology was affected in *Sox10-Cre;Lmx1b* ^*fl/fl*^ mutants. Therefore, we re-examined the embryonic origin of the coronal suture at multiple positions along the apical-basal axis (Figure 2). We found that the apical part of the coronal suture mesenchyme was normally neural crest-derived (Figures 2D, G, M) even though most of the coronal suture was mesoderm-derived as previously reported (Jiang et al., 2002; Yoshida et al., 2008). This explained the lack of fusion in the apical coronal suture in *Mesp1-Cre;Lmx1b* ^*fl/fl*^ mutants, as the suture mesenchyme here was made of cells still expressing *Lmx1b* (Figure 2J; *n* = 3). In contrast, the basal part of the coronal suture was patent in *Mesp1-Cre;Lmx1b* ^*fl/fl*^ mutants despite being mesoderm-derived (Figure 2L; *n* = 3). In *Sox10-Cre;Lmx1b* ^*fl/fl*^ mutants, the entire coronal suture, including the apical part, was made of mesoderm-derived cells, which also formed the osteogenic front of the frontal bone (Figure 2O–P; *n* = 3). However, the contribution of the mesodermal cells was only at the posterior tip of the frontal bone (arrow in Supplementary Figures S4B, D, F), and thus the overall composition of the frontal bone did not change significantly in *Sox10-Cre;Lmx1b* ^*fl/fl*^ mutants (Supplementary Figure S4G).

We examined the origin of the lambdoidal suture because it was not clearly determined in earlier studies. At P0, the lambdoidal suture mesenchyme was entirely from the mesoderm lineage, whether it abutted the medial part of the interparietal bone, which is neural crest-derived (Supplementary Figures S5D, G), or the lateral part of the interparietal bone, which is mesoderm-derived (Supplementary Figures S5E, F, H, I). A previous study reported presence of neural crest-derived cells in the lambdoidal suture at P7 (Behr et al., 2011), and thus the composition may change over time.

### 3.4 Inactivation of *Lmx1b* leads to widespread osteogenesis in the cranial mesenchyme from early stages of calvarial development, but not over the midbrain

**FIGURE 3:**
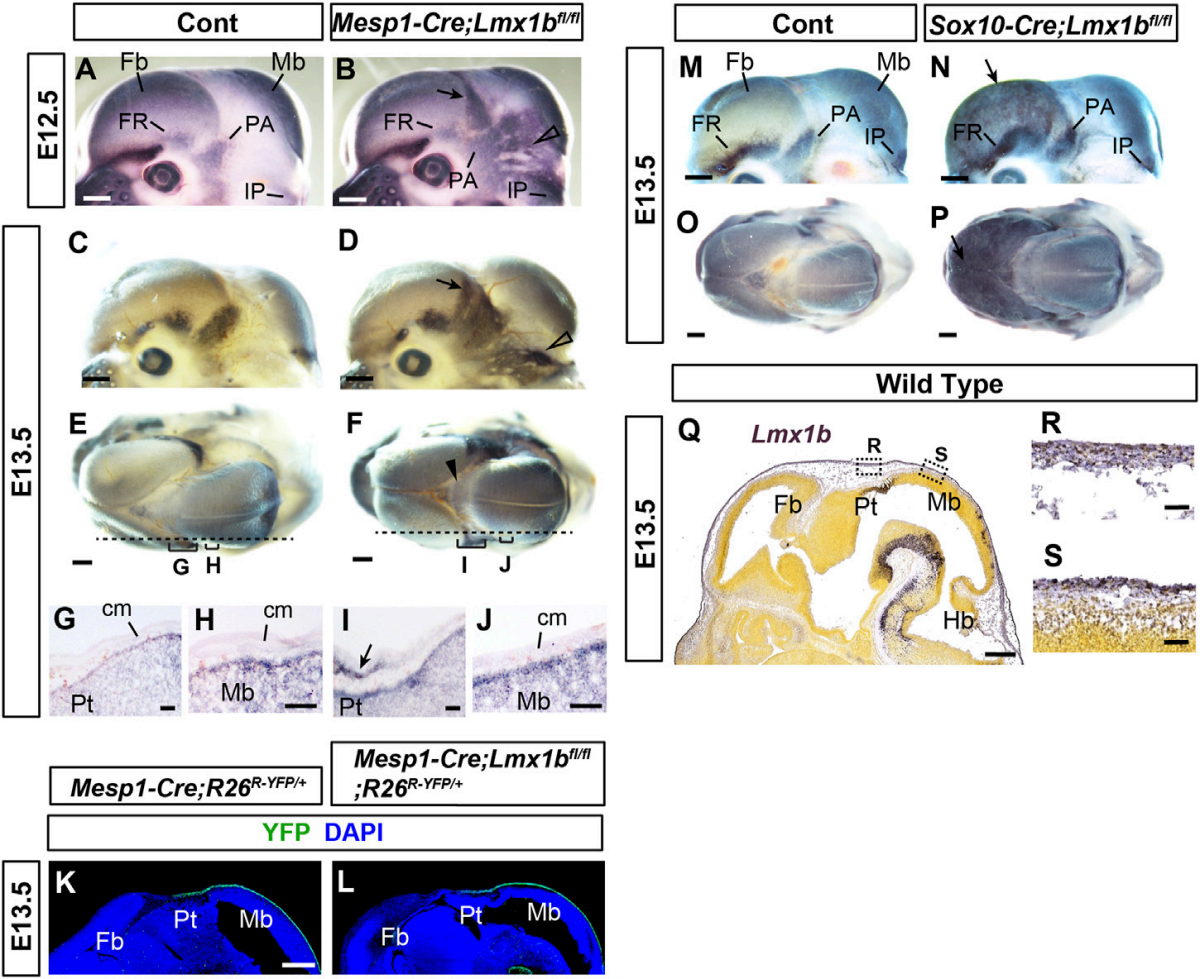
Early defects in the calvarial development in mesoderm-specific and neural crest-specific *Lmx1b* deletion mutants **(A–F, M–P)** Mouse embryo heads processed by whole-mount alkaline phosphatase staining. **(E, F, O, P)** are superior views, and the rest are lateral views. The arrows in **(B, D, N, P)** point to heterotopic osteogenesis in the apical cranial mesenchyme of the *Lmx1b* mutants. The open arrowheads in **(B, D)** point to the heterotopic osteogenesis in the posterior cranial mesenchyme. The arrowhead in F points to barely detectable alkaline phosphatase staining at the dorsal midline. For **(A–F)**, 2 controls and 4 mutants (E12.5) and 6 controls and 4 mutants (E13.5) were examined. For **(M–P)**, 8 controls and 2 mutants were examined. **(G–J)** Sagittal sections of the heads processed by alkaline phosphatase staining, showing the positions equivalent to the dotted lines and the brackets in **(E, F)**. The arrow in I points to the heterotopic osteogenesis in the mesenchyme overlying the pretectum (Pt) at the forebrain-midbrain junction. 2 controls and 2 mutants were examined. **(K, L)** Sagittal sections of the heads at an equivalent plain of section to **(G–J)** stained for YFP. 4 controls and 3 mutants were examined. **(Q–S)** From Allen Mouse Brain Atlas (AIBS, 2004), specimen 320-3462. A sagittal section of the head at an equivalent plain of section to **(G–L)** processed by RNA *in situ* hybridization for *Lmx1b*. All the controls in this figure are *Lmx1b* ^*fl/fl*^ or *Lmx1b* ^*fl/+*^ unless indicated otherwise. Bar in **(A–F, K–Q)**: 0.5 mm, bar in **(G–J, R, S)**: 0.05 mm cm, cranial mesenchyme; Fb, forebrain; Hb, hindbrain; Mb, midbrain; Pt, pretectum.

To characterize the primary changes underlying the calvarial phenotypes of *Mesp1-Cre;Lmx1b* ^*fl/fl*^ and *Sox10-Cre;Lmx1b* ^*fl/fl*^ mutants, we examined early stages of calvarial development using alkaline phosphatase as a marker of osteogenesis (Figures 3A–J, M–P). At E12.5 (*n* = 4) and E13.5 (*n* = 6), *Mesp1-Cre;Lmx1b* ^*fl/fl*^ mutants showed widespread osteogenesis in the posterior half of the cranial mesenchyme (arrows and open arrowheads in Figures 3B, D, I). However, the aberrant alkaline phosphatase expression was weak at the dorsal midline in *Mesp1-Cre;Lmx1b* ^*fl/fl*^ mutants (arrowhead in Figure 3F). More importantly, it was absent from the mesenchyme over most of the midbrain, the position of the future posterior fontanel (Figures 3A–D). This spatial restriction in osteogenesis was clear at E12.5, when the bulging of the midbrain was not yet evident in the mutants (Figures 3A, B). Besides, the brain is not where primary defects are expected from *Mesp1-Cre*-mediated mutation. Thus, the bulging of the brain at the posterior fontanel is likely to be a consequence, rather than the cause, of the mesenchyme over the midbrain being an 'island’ of soft spot encircled by bone. We confirmed that *Mesp1-Cre* had been active in this region of the mesenchyme in *Mesp1-Cre;Lmx1b* ^*fl/fl*^ mutants (Figures 3K, L) (*n* = 3). Unlike *Mesp1-Cre;Lmx1b* ^*fl/fl*^ mutants, *Sox10-Cre;Lmx1b* ^*fl/fl*^ mutants showed alkaline phosphatase staining covering the entire forebrain (arrows in Figures 3N, P; *n* = 2). Of note, *Lmx1b* expression during normal development appeared similar in the cranial mesenchyme over different parts of the brain (Figures 3Q–S).

### 3.5 *Lmx1b* in the neural crest-derived mesenchyme, but not the mesoderm-derived mesenchyme, has a non-cell autonomous effect on coronal suture development

**FIGURE 4:**
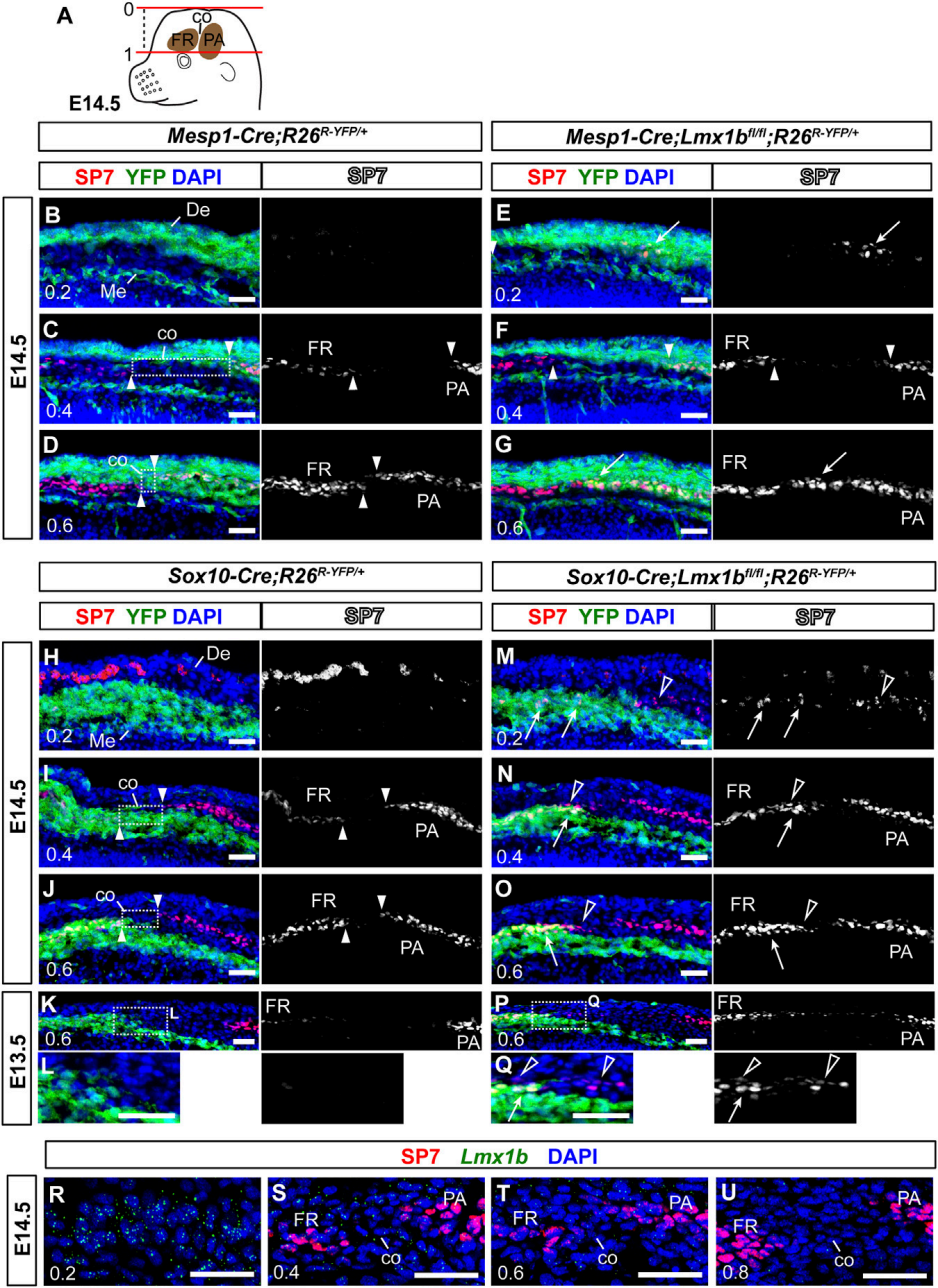
Non-cell autonomous effect on coronal suture development from neural crest-specific deletion of *Lmx1b* **(A)** A schematic of an E14.5 embryo head explaining the numerical designation of the position of transverse sections along the apical-basal axis. The top of the head is 0, and the top of the eye is 1. See Materials and Methods Section 2.3 for details. **(B–Q)** Transverse sections processed by immunofluorescence for SP7 and YFP. Each of **(B–D, E–G, H–J, M–O)** sets are an apical-to-basal series of three sections from one embryo, and the numbers in the bottom-left corner of each panel indicate the position of the section. **(L, Q)** are enlargements of the boxed areas in **(K, P)**, respectively. The arrowheads point to the ends of the frontal bone (FR) and the parietal bone (PA), and the boxes in **(C, D, I, J)** indicate prospective coronal suture mesenchyme (co) between the two osteogenic fronts. The arrows in **(E, G, M–Q)** point to heterotopic or enhanced osteogenesis of *Lmx1b*-mutant cells (YFP^+^). The open arrowheads in **(M–Q)** point to mesoderm-derived cells undergoing aberrant osteogenesis after neural crest-specific deletion of *Lmx1b*. The red spots in the dermis (De) in **(H, M)** are auto-fluorescence from blood cells. 3 samples per genotype were examined at E14.5, and 2 samples per genotype were examined at E13.5. **(R–U)** An apical-to-basal series of transverse sections of an E14.5 control embryo (*Sox10-Cre;R26* ^*R-YFP/+*^) head processed by fluorescence RNA *in situ* hybridization for *Lmx1b* and immunofluorescence for SP7. *Lmx1b* signal was captured in far-red but pseudo-colored to green. Two control embryos were examined. Bar: 0.05 mm. De, dermis; Me, meninges.

Although we found neural crest-derived cells in the apical part of the coronal suture at P0 (Figure 2), this could have been due to the coronal suture merging with the neural crest-derived sutures at the dorsal midline late in gestation, rather than due to coronal suture progenitors arising from the neural crest-derived mesenchyme during early development. Therefore, we examined the coronal suture at E14.5 to clarify the contribution of the neural crest lineage (Figure 4). To identify matching sections across different samples consistently, we labeled apical-basal positions using a numerical system in which the top of the head was 0 and the top of the eye was 1 (Figure 4A; see Materials and Methods Section 2.3 for details). We were able to discern the prospective coronal suture flanked by the frontal bone and the parietal bone rudiments up to 0.4 position (Figures 4C, I). Here, we found that the presumptive coronal suture progenitors were mostly neural crest-derived (YFP^−^ in the box in Figure 4C and YFP^+^ in the box in Figure 4I), whereas they were mesoderm-derived at more basal positions (YFP^+^ in the box in Figure 4D and YFP^−^ in the box in Figure 4J).

In E14.5 *Mesp1-Cre;Lmx1b* ^*fl/fl*^ mutants (*n* = 3), SP7 was detected at an apical position (0.2) where it is not detected normally (arrow in Figure 4E), and also in what should be mesoderm-derived coronal suture progenitors at 0.6 (arrow in Figure 4G). However, there was no evidence of aberrant osteogenesis in the neural crest-derived cells in these mutants (Figures 4B–G). In contrast, *Sox10-Cre;Lmx1b* ^*fl/fl*^ mutants showed ectopic or enhanced expression of SP7 not only in the neural crest-derived cells (arrows in Figures 4M–O, Q) but also in the adjacent mesoderm-derived cells (open arrowheads in Figures 4M–O, Q; *n* = 3 for E14.5, *n* = 2 for E13.5). For example, the mesodermal cells bordering the neural crest-derived mesenchyme at 0.6 (YFP^−^ cells in the boxes in Figures 4J, K, P) does not normally express SP7 (Figures 4J, K, L), but they did in *Sox10-Cre;Lmx1b* ^*fl/fl*^ mutants (open arrowheads in Figures 4O, Q). This is a non-cell autonomous phenotype because the Cre was not active the mesoderm. Still, the coronal suture was able to form in these mutants, just a few cells removed from the neural crest-mesoderm boundary (Figures 4N, O). Consistent with the observation at P0 (Figure 2), the coronal suture progenitors at 0.4 were from the mesoderm, instead of the neural crest, in *Sox10-Cre;Lmx1b* ^*fl/fl*^ mutans (Figure 4N), indicating that cells lacking *Lmx1b* expression were unable to contribute to the coronal suture.

*Lmx1b* expression in the coronal suture had not been described before, and thus we examined it at various positions along the apical-basal axis during normal development (Figures 4R–U). As previously reported on coronal sections (Cesario et al., 2018), *Lmx1b* was expressed predominantly around the top of the head (0.2) while absent from the supra-orbital mesenchyme (0.8) (Figures 4R, U). In between, low levels of *Lmx1b* mRNA were detected in the coronal suture progenitors at 0.4 and 0.6 (Figures 4S, T).

### 3.6 Neural crest-specific overexpression of *Lmx1b* uncouples the coronal suture from the lineage boundary in the basal part, but not in the apical part

To confirm the non-cell autonomous function of LMX1B, we overexpressed it in the neural crest-derived cells using *Sox10-Cre* and a *ROSA26* knockin allele with a floxed stop cassette followed by *Lmx1b* coding sequence (*R26* ^*Lmx1b/+*^) (Li et al., 2010).

**FIGURE 5:**
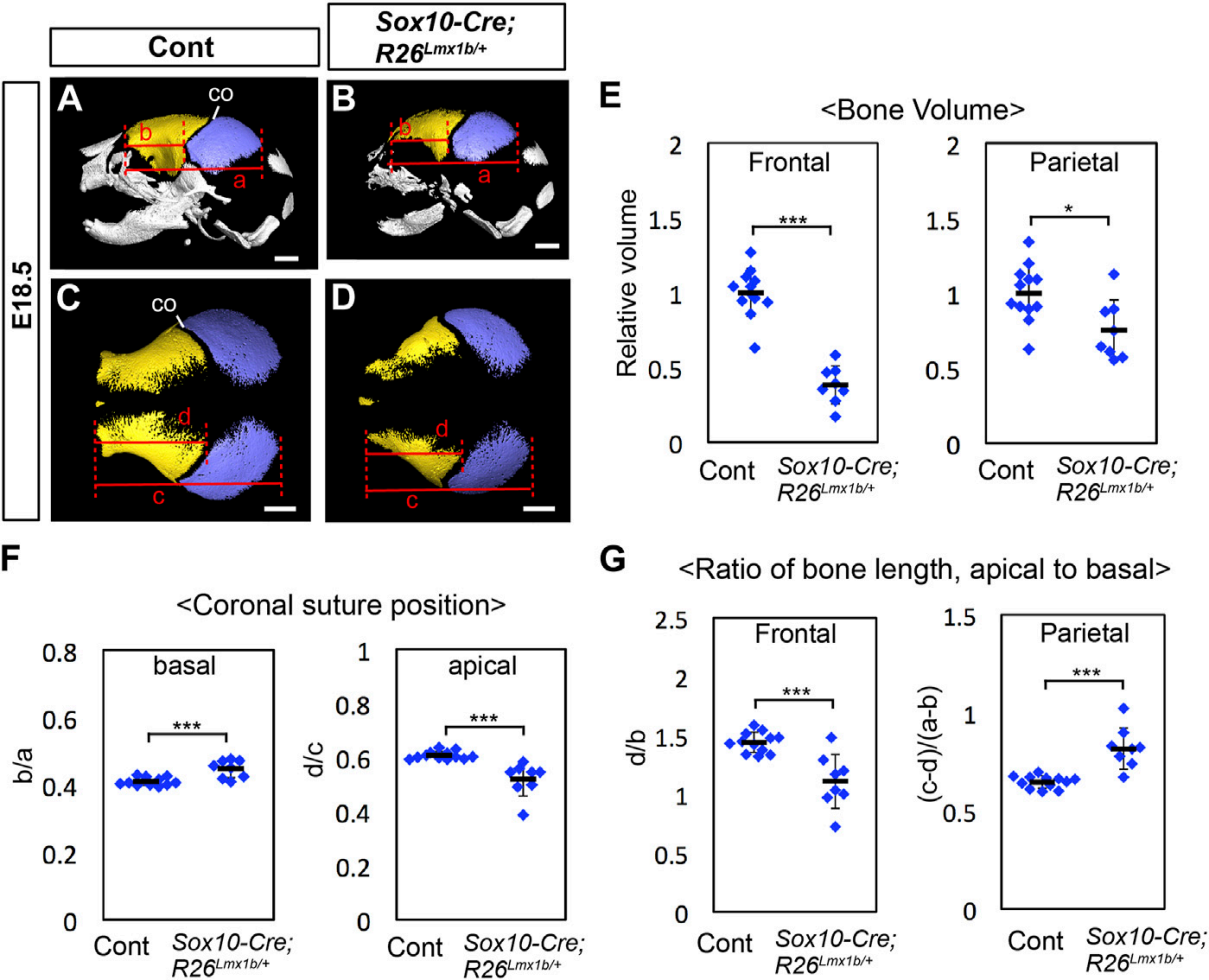
Calvarial phenotype of neural crest-specific *Lmx1b* overexpression mutants **(A–D)** 3D reconstruction from microCT scans of E18.5 skulls. Yellow: frontal bone, blue: parietal bone. **(A, B)** are lateral views of the whole skull. **(C, D)** are superior views of the frontal bone and the parietal bone only. 12 controls (=*Sox10-Cre*) and 8 mutants were examined. **(E)** Volumes of the frontal bone and the parietal bone measured from segmentations in **(A–D)** and normalized to the average volume from control samples (= 1). **(F)** Relative position of the coronal suture (co) measured as a ratio of b to a in **(A, B)** (basal) or a ratio of d to c in **(C, D)** (apical). The landmarks used for the measurements are the same as those for Figure 1AC. **(G)** Ratio of the antero-posterior bone lengths measured at the level of the apical end of the coronal suture **(C, D)** and at the level of the basal end of the coronal suture **(A, B)**. Bar: 1 mm *: *p* < 0.05, ***: *p* < 0.001.

At E18.5, the frontal bone volume was reduced by more than 50% in *Sox10-Cre;R26* ^*Lmx1b/+*^ mutants compared with controls (Figures 5A–E; *n* = 8). The parietal bone was also reduced by ∼25% on average, as expected from a non-cell autonomous effect of *Lmx1b* overexpression. We also examined the relative position of the coronal suture within the frontal bone-coronal suture-parietal bone unit in *Sox10-Cre;R26* ^*Lmx1b/+*^ mutants (Figures 5A–D, F). At the apical end, the mutant coronal suture shifted in the anterior direction compared with controls, consistent with the frontal bone being more severely reduced than the parietal bone (Figures 5C, D, F). Surprisingly, the basal end of the coronal suture shifted posteriorly in these mutants (Figures 5A, B, F). Examination of the frontal bone and the parietal bone separately showed that they were not uniformly affected along the apical-basal axis in the mutants. The frontal bone was preferentially reduced in the apical part, whereas the parietal bone was preferentially reduced in the basal part (Figure 5G).

**FIGURE 6:**
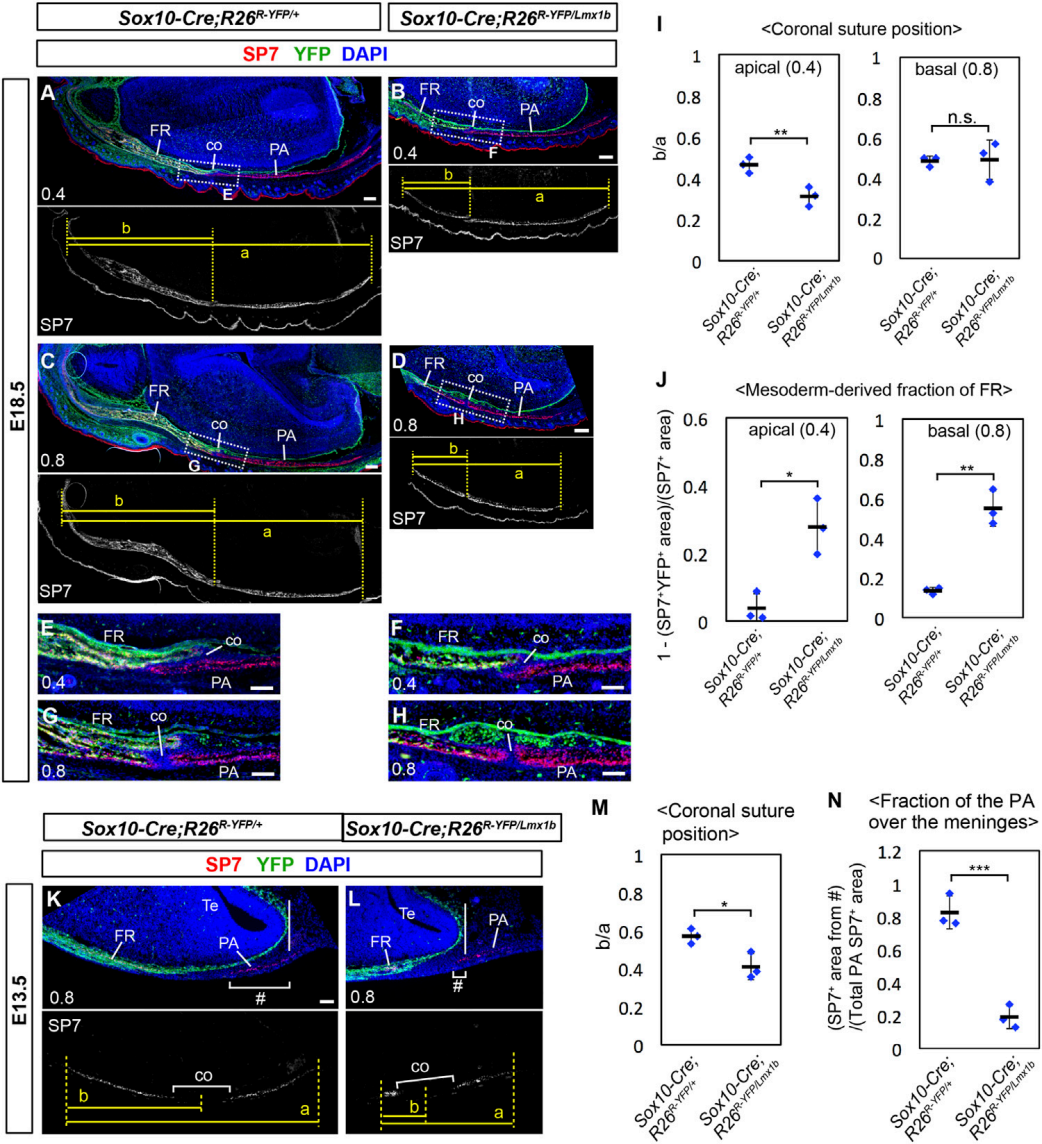
Coronal suture phenotype of *Lmx1b* overexpression mutants. **(A–H, K, L)** Transverse sections processed by immunofluorescence for SP7 and YFP. The numbers in the bottom-left corner of each panel indicate the position of the section as defined in Figure 4A. The boxes in **(A–D)** mark the areas shown in **(E–H)**, respectively. 3 samples per genotype were examined. **(I)** Relative position of the coronal suture (co) measured as a ratio of b to a in **(A–D)**. The dotted lines through landmarks were drawn perpendicular to the anterior-posterior axis of the head, and the distance between the two dotted lines were measured. The landmarks for each measurement are a: from the anterior end of the frontal bone to the posterior end of the parietal bone, b: from the anterior end of the frontal bone to the midpoint of the bone overlap at the coronal suture. **(J)** Mesoderm-derived fraction of the frontal bone. The neural crest-derived fraction was measured as described in Supplementary Figure S4, and then subtracted from 1. **(M)** Relative position of the coronal suture measured as a ratio of b to a in **(K, L)**. The landmarks for each measurement are a: from the anterior end of the frontal bone to the posterior end of the parietal bone, b: from the anterior end of the frontal bone to the midpoint of the prospective coronal suture. **(N)** Fraction of the parietal bone over the telencephalon meninges, calculated by dividing SP7^+^ area within # brackets in **(K, L)** with total SP7^+^ area of the parietal bone rudiment. The solid vertical lines in **(K, L)** mark the posterior end of the telencephalon meninges. Bar in **(A–D)**: 0.2 mm, bar in **(E–L)**: 0.1 mm. Te: telencephalon. *: *p* < 0.05, **: *p* < 0.01, ***: *p* < 0.001, n.s.: not significant (*p* > 0.05).

To better understand the coronal suture phenotype of *Sox10-Cre;R26* ^*Lmx1b/+*^ mutants, we examined the transverse sections of the heads in which YFP labeled the neural crest-derived cells (Figure 6). The measurements from perinatal samples confirmed that the apical part of the coronal suture, but not the basal part, shifted to the anterior direction in the mutants (Figures 6A–D, I; *n* = 3). The mutant frontal bone contained a larger fraction of mesoderm-derived cells (YFP^−^) than controls (Supplementary Figure S6; Figures 6A–H, J). In the apical part (0.4), these cells were broadly scattered, and the coronal suture more or less coincided with the neural crest-mesoderm boundary (Figures 6E, F). In contrast, in the basal part (0.8), the posterior half of the mutant frontal bone was almost entirely made of the mesoderm cells, and the coronal suture was located inside the mesoderm domain (Figures 6G, H, J).

We examined how the initial osteogenesis pattern was affected in *Sox10-Cre;R26* ^*Lmx1b/+*^ mutants at E13.5. Consistent with the phenotype at E18.5, both the frontal bone and the parietal bone rudiments were reduced in the mutants (*n* = 3). However, throughout the apical-basal axis, the frontal bone rudiment was more severely affected than the parietal bone rudiment, and thus the prospective coronal suture showed an anterior shift (Supplementary Figure S7; Figures 6K–M). Therefore, the early phenotype was similar to that of the apical part at E18.5, which suggested that the basal part had undergone a patterning change in *Sox10-Cre;R26* ^*Lmx1b/+*^ mutants later in development. We also noted that *Lmx1b* overexpression affected the parietal bone formation preferentially in the mesenchyme overlying the forebrain meninges, such that this region (# in Figures 6K, L) contained most of the parietal bone rudiment in control embryos, but less than half in the mutant embryos (Figure 6N).

## 4 Discussion

**FIGURE 7:**
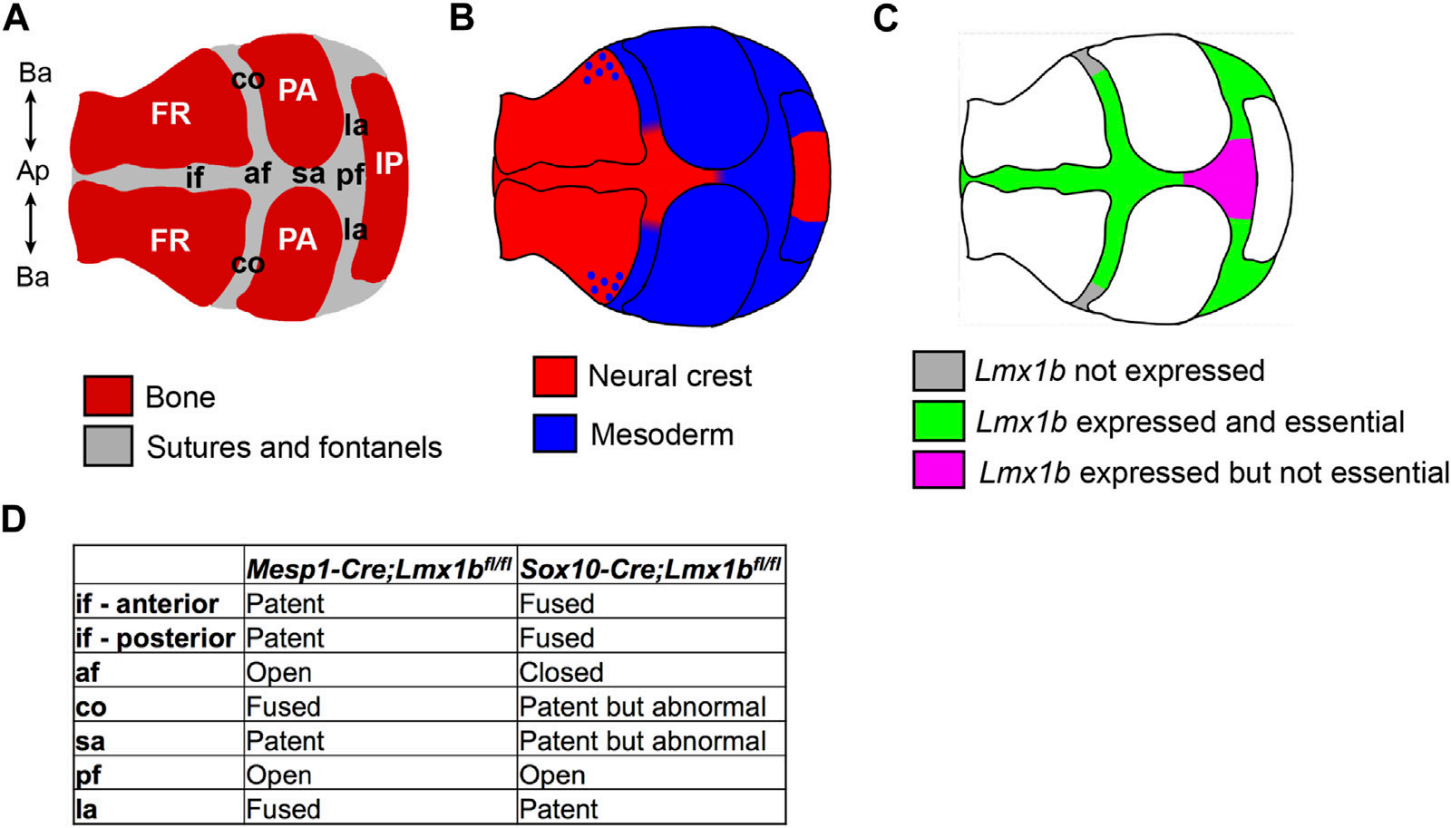
Summary of the embryonic origin and *Lmx1b* function in different regions of the calvaria. Schematics showing the components of the calvaria **(A)**, revised map of the lineage contribution **(B)**, and the region-specific requirement for *Lmx1b* to inhibit osteogenesis **(C)**. **(D)** Comparison of the suture phenotypes between mesoderm-specific and neural crest-specific *Lmx1b* deletion mutants. af, anterior fontanel; Ap-Ba, apical-basal axis of the calvaria; co, coronal suture; FR, frontal bone; if, interfrontal suture; IP, interparietal bone; la, lambdoidal suture; PA, parietal bone; pf, posterior fontanel; sa, sagittal suture.

In this study, we have clarified a spatial specificity of *Lmx1b* function by analyzing lineage-specific deletion and overexpression mutants. In the calvaria, *Lmx1b* was essential to prevent osteogenesis in all sutures except for the basal part of the coronal suture, the posterior part of the sagittal suture, and the posterior fontanel (Figure 7). Furthermore, *Lmx1b* in the neural crest-lineage, but not the mesoderm lineage, had a non-cell autonomous effect at the coronal suture. In addition, although *Lmx1b* was expressed in the facial mesenchyme, it did not play the same crucial role as in the calvaria. Together, our findings on *Lmx1b* highlight the regional difference in genetic regulation of osteogenesis during development, even between physically and/or ontogenically very close areas of the mesenchyme. More importantly, a couple of unexpected phenotypes of the *Lmx1b* mutants gave us new insights into calvarial development.

### 4.1 Embryonic origin of the calvarial sutures

From what had been known about the embryonic origin of different components for the calvaria, we expected that the interfrontal suture and the sagittal suture would be lost in *Sox10-Cre;Lmx1b* ^*fl/fl*^ mutants. However, the sagittal suture was patent in these mutants, which led us to discover that it normally comprised both neural crest-derived and mesoderm-derived cells. Also, the complex coronal suture phenotype in *Mesp1-Cre;Lmx1b* ^*fl/fl*^ mutants revealed that the coronal suture has three distinct segments along the apical-basal axis with respect to the embryonic origin and regulation by *Lmx1b*: neural crest-derived and *Lmx1b*-dependent, mesoderm-derived and *Lmx1b*-dependent, mesoderm-derived and *Lmx1b*-independent (Figure 7). A previous study reported neural crest contribution to the coronal suture, but it was only mentioned as a part of the periosteum and the dura mater (Doro et al., 2019), not the suture mesenchyme proper as we found. More importantly, all the earlier reports of lineage analyses showed whole mount samples, where it is difficult to distinguish overlapping layers of tissue, and/or sections from only one position for each suture (Jiang et al., 2002; Yoshida et al., 2008; Behr et al., 2011; Doro et al., 2019). The intra-suture heterogeneity described here indicates that a careful attention to the precise position is necessary when examining suture phenotypes at a high resolution.

According to our results, *Lmx1b* was essential in all the neural crest-derived suture areas, but it was dispensable in some of the mesoderm-derived suture areas (Figure 7). It is possible that the embryonic origin of the cells is important for the role of *Lmx1b* in suture development. Alternatively, different positions of the suture cells within the calvaria may determine their dependence on *Lmx1b*, as discussed below.

In *Sox10-Cre;Lmx1b* ^*fl/fl*^ mutants, the neural crest-derived cells occupying normal positions of the interfrontal suture and the anterior sagittal suture became the heterotopic bone at the midline (Supplementary Figure S3). Similarly, we believe that the neural crest-derived cells supposed to make the apical coronal suture mesenchyme were incorporated into the frontal bone, contributing to its posterior extension (reflected in the posterior displacement of the apical coronal suture). However, this is difficult to prove because the frontal bone is normally made of the neural crest-derived cells, those migrating from the supra-orbital mesenchyme (Jiang et al., 2002; Yoshida et al., 2008).

### 4.2 Establishing the sutures along the dorsal midline of the calvaria

It is incompletely understood how positions of the bone and the sutures are determined during calvarial development. Regulatory factors for calvarial patterning likely include prepatterning of the cranial mesenchyme by region-specific gene expression, environmental cues via secreted signaling molecules from the neighboring tissue, and localized accumulation of mechanical strain produced by the growing brain. Once osteogenesis is initiated at certain positions, the pattern is enforced by a reaction-diffusion mechanism involving activators and inhibitors from the nascent osteogenic centers (Lee et al., 2019; Galea et al., 2021). There may be a hierarchy among these factors, and potentially a difference in their relative importance depending on the exact location.

A recent study suggested that a difference in the volumetric strain from the brain was the reason why the osteogenic centers appeared in the baso-lateral region of the head but not at the apex during normal development (Lee et al., 2019). However, ossification at the interfrontal suture, the anterior fontanel, and the anterior sagittal suture in *Sox10-Cre;Lmx1b* ^*fl/fl*^ mutants indicates that a superseding factor at this location is intrinsic patterning of the cranial mesenchyme by way of differential expression of *Lmx1b*. Furthermore, a suture-like gap appeared between the heterotopic midline bone and the endogenous frontal bone, most likely due to mutual inhibition via the reaction-diffusion mechanism (Galea et al., 2021), but this was also abrogated later in the absence of *Lmx1b* (Figure 1).

In contrast, there seems to be an *Lmx1b*-independent mechanism to prevent osteogenesis at the posterior sagittal suture and the posterior fontanel, based on the results from *Mesp1-Cre;Lmx1b* ^*fl/fl*^ and *Prrx1-Cre*;*Lmx1b* ^*fl/-*^ mutants (Figure 1; Cesario et al., 2018). Notably, *Prrx1-Cre*-mediated deletion of *Gnas*, encoding a component of G protein-coupled receptor signaling, led to heterotopic ossification at the dorsal calvaria preferentially affecting the posterior fontanel (Xu et al., 2018). *Gnas* is ubiquitously expressed during development (Magdaleno et al., 2006), and thus it is not clear why the anterior and the posterior regions of the calvaria have opposite patterns of dependence on *Lmx1b* and *Gnas* to prevent osteogenesis. We speculate that a difference in interactions between the suture and different regions of the brain (forebrain vs. midbrain) might be involved.

### 4.3 Determining the position of the coronal suture

Because the frontal bone, the coronal suture, and the parietal bone arise from one continuous and seemingly homogenous block of mesenchyme, it is particularly intriguing how the suture position is determined within this unit. An interesting phenotype of *Mesp1-Cre;Lmx1b* ^*fl/fl*^ mutants was that the apical part and the basal part of the coronal suture were completely disjointed. This result suggested that the positions of the two segments could be determined independently of each other, although it does not necessarily mean that this is the case during normal development.

How we analyzed the relative position of the coronal suture in *Lmx1b* mutants is very similar to what others did for lineage-specific mutants of two craniosynostosis genes, *Twist1* and *Tcf12* (measuring the ratio between the lengths of the sagittal suture and the interfrontal suture) (Teng et al., 2018; Ting et al., 2022), except that we analyzed the apical end and the basal end separately. A common finding from all three studies is that the apical coronal suture position at perinatal or postnatal stages closely reflects the pattern of osteogenesis at early stages (E13.5 or earlier) determined by genetic regulation intrinsic to the cranial mesenchyme. When a mutation enhanced osteogenesis in the neural crest-derived mesenchyme, the coronal suture was displaced posteriorly. When a mutation enhanced osteogenesis in the mesoderm-derived mesenchyme, the coronal suture shifted anteriorly. *Tcf12* and *Twist1* were shown to regulate cell proliferation, and the displacement of the coronal suture in these mutants was attributed to differential growth of the frontal bone vs. the parietal bone (Teng et al., 2018). However, our earlier study did not find a significant effect of *Lmx1b* mutation on cell proliferation (Cesario et al., 2018). Therefore, the phenotype of *Lmx1b* mutants likely reflects mis-patterning of the cranial mesenchyme at the beginning of the calvarial development.

As to the position of the basal end of the coronal suture, we did not find a significant change in *Sox10-Cre;Lmx1b* ^*fl/fl*^ or *Mesp1-Cre;Lmx1b* ^*fl/fl*^ mutants. This was consistent with the fact that *Lmx1b* is normally not expressed in the supra-orbital mesenchyme, which contains the base of the coronal suture. However, in *Sox10-Cre;R26* ^*Lmx1b/+*^ mutants, *Lmx1b* was overexpressed even in the supra-orbital mesenchyme, and thus we expected an anterior shift of the coronal suture associated with the frontal bone deficiency at the basal end as well as at the apical end. Instead, we found that the mesoderm-derived cells partially compensated for the frontal bone deficiency between E13.5 and E18.5, apparently at the expense of the parietal bone growth in the basal part (Figures 5, 6). As a result, the anterior shift of the coronal suture at E13.5 was “rectified” by E18.5 (in fact, over-corrected to the posterior shift), and in the process, the coronal suture became separated from the neural crest-mesoderm boundary. Although we do not know the molecular and cellular changes underlying this phenotype, it suggests that an unknown factor overrode the early pattern of osteogenesis in the cranial mesenchyme during the course of development. In addition, the phenotype revealed that being at the lineage boundary was not essential for the basal coronal suture to be maintained, although it might be important for initial specification.

It is unclear why the positions of the apical end and the basal end of the coronal suture might be regulated differently. One potential factor is that the basal part of the calvaria, but not the apical part, develops in close association with the chondrocranium, which may influence development of the calvarial bone and sutures (Motch Perrine et al., 2022).

While we found aberrant contribution of the mesoderm-derived cells to the frontal bone in both *Sox10-Cre;Lmx1b* ^*fl/fl*^ and *Sox10-Cre;R26* ^*Lmx1b/+*^ mutants, the mechanisms involved are most likely different between the two. In *Sox10-Cre;Lmx1b* ^*fl/fl*^ mutants, SP7 expression was activated in the mesodermal cells next to the neural crest-derived domain from an early stage (E13.5) (Figure 4), and this phenotype remained restricted to the lineage boundary at P0 (Supplementary Figure S4). Therefore, we believe that there was an error in mesodermal cell fate specification regulated by *Lmx1b* via short-range secreted signaling molecules (see Section 4.4 for details). In contrast, in *Sox10-Cre;R26* ^*Lmx1b/+*^ mutants, there was a large scale change in the frontal bone composition accompanied by a significant modification in the calvarial bone pattern between E13.5 and E18.5 (Figure 6). Thus, we speculate that the incorporation of the mesodermal cells to the frontal bone is due to an adjustment secondary to the early defect, as discussed above, rather than due to regulation of mesoderm osteogenesis by *Lmx1b*.

We did not pursue *Mesp1-Cre*-mediated overexpression of *Lmx1b* because *Mesp1-Cre* is active in the cardiovascular system including the capillary endothelial cells throughout the body (Saga et al., 1999). Consequently, overexpressing *Lmx1b* with *Mesp1-Cre* is likely to cause a global disruption in the embryo, which in turn would complicate analysis of the phenotype. Hypothetically, overexpression of *Lmx1b* in the mesoderm-derived calvarial osteoprogenitors is expected to impair parietal bone formation, possibly with partial compensation by neural crest-derived cells. However, we are cautious with making predictions considering that we have encountered many surprising phenotypes in the current study.

### 4.4 Non-cell autonomous function of *Lmx1b*

We found that *Lmx1b* deletion in the neural crest-derived cells, but not in the mesoderm-derived cells, led to non-cell autonomous induction of osteogenesis at the coronal suture (Figure 4). In *Sox10-Cre;Lmx1b* ^*fl/fl*^ mutants, osteogenesis was enhanced in the neural crest-derived cells, judging from SP7 expression. More importantly, SP7 was also induced in the adjacent mesoderm-derived cells even though *Lmx1b* was not deleted in them. In the coronal suture region, the main differences between the contributions from the neural crest and the mesoderm are that only the neural crest-derived cells make the fibroblasts of the forebrain meninges underneath the suture, while only the mesoderm-derived cells make the capillary endothelial cells (Le Douarin and Kalcheim, 1999). Therefore, we hypothesize that the non-cell autonomous effect of *Lmx1b* involves its expression in the meninges (Dasgupta et al., 2019). This is consistent with the phenotype in *Sox10-Cre;R26* ^*Lmx1b/+*^ mutants, in which the parietal bone development was preferentially inhibited in the mesenchyme over the forebrain meninges.

It has been known for decades that the meninges regulate the sutures, although most studies focused on suture maintenance at late fetal or postnatal stages (Grova et al., 2012; Dasgupta and Jeong, 2019). The meninges express diverse secreted signaling molecules including members of transforming growth factor β (TGFβ), fibroblast growth factor (FGF), bone morphogenetic protein (BMP), Chemokine (C-X-C motif) ligand, and retinoic acid (Dasgupta and Jeong, 2019), and one or more of them may be regulated by *Lmx1b*.

However, the non-cell autonomous mode is clearly not the major mechanism for the function of *Lmx1b*. The coronal suture was able to form in *Sox10-Cre;Lmx1b* ^*fl/fl*^ mutants, and the parietal bone eventually grew over the forebrain meninges in *Sox10-Cre;R26* ^*Lmx1b/+*^ mutants. Therefore, we conclude that *Lmx1b* regulates the fate of cranial mesenchyme cells mainly via a cell-autonomous mechanism, which is common for a transcription factor. Understanding the exact mechanism of LMX1B function awaits identification of its transcriptional targets. Nevertheless, our finding suggests a potential novel role for *Lmx1b* in regulating development of the meninges.

## Data Availability

The original contributions presented in the study are included in the article/Supplementary Materials, further inquiries can be directed to the corresponding author.
