# Supplementary material for: Lineage-specific mutation of Lmx1b provides new insights into distinct regulation of suture development in different areas of the calvaria
Source: Front Physiol. 2023 Aug 1;14:1225118. doi: 10.3389/fphys.2023.1225118 (PMC10427921; doi:10.3389/fphys.2023.1225118)
Supplement: Supplementary file 1 [file DataSheet1.PDF]

Supplementary Material

Lineage-specific mutation of *Lmx1b* provides new insights into distinct regulation of suture development in different areas of the calvaria

Angel Cabrera Pereira[1,†], Krishnakali Dasgupta[1,†], Thach-Vu Ho[2], Maria Pacheco-Vergara[1], Julie Kim[1], Niam Kataria[1], Yaowei Liang[1], Jeslyn Mei[1,3], Jinyeong Yu[1,4], Lukasz Witek[1], Yang Chai[2], and Juhee Jeong[1,*]*

Correspondence: Juhee Jeong: jj78@nyu.edu

Supplementary Figures

Figure S1

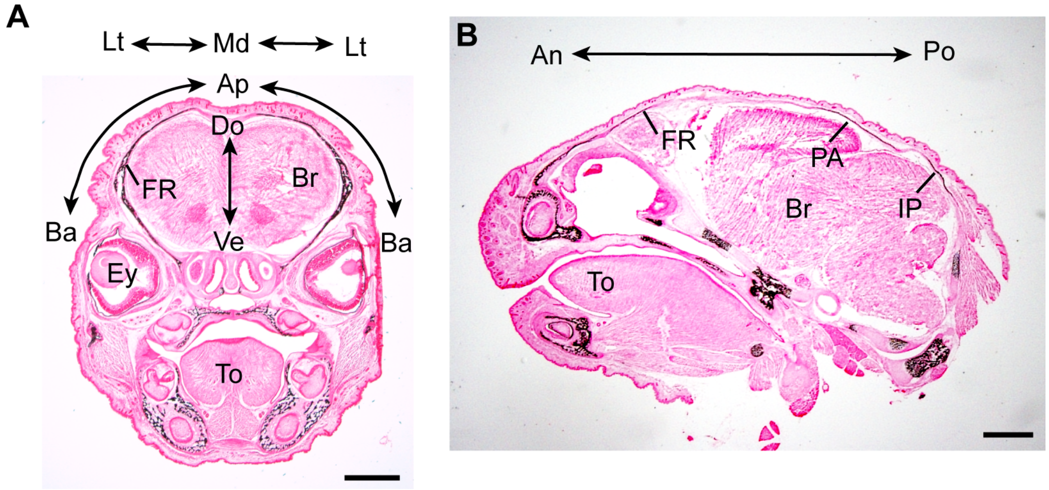

Supplementary Figure 1. Definitions of axes in the head

(A,B) Coronal (A) and sagittal (B) sections of the head of normal E18.5 embryos stained with silver nitrate for bone (dark brown) and nuclear fast red. Bar: 1 mm. An-Po: anterior-posterior axis, Ap-Ba: apical-basal axis, Do-Ve: dorsal-ventral axis, Md-Lt: medial-lateral axis, Br: brain, Ey: eye, FR: frontal bone, IP: interparietal bone, PA: parietal bone, To: tongue.

frontiers

Figure S2

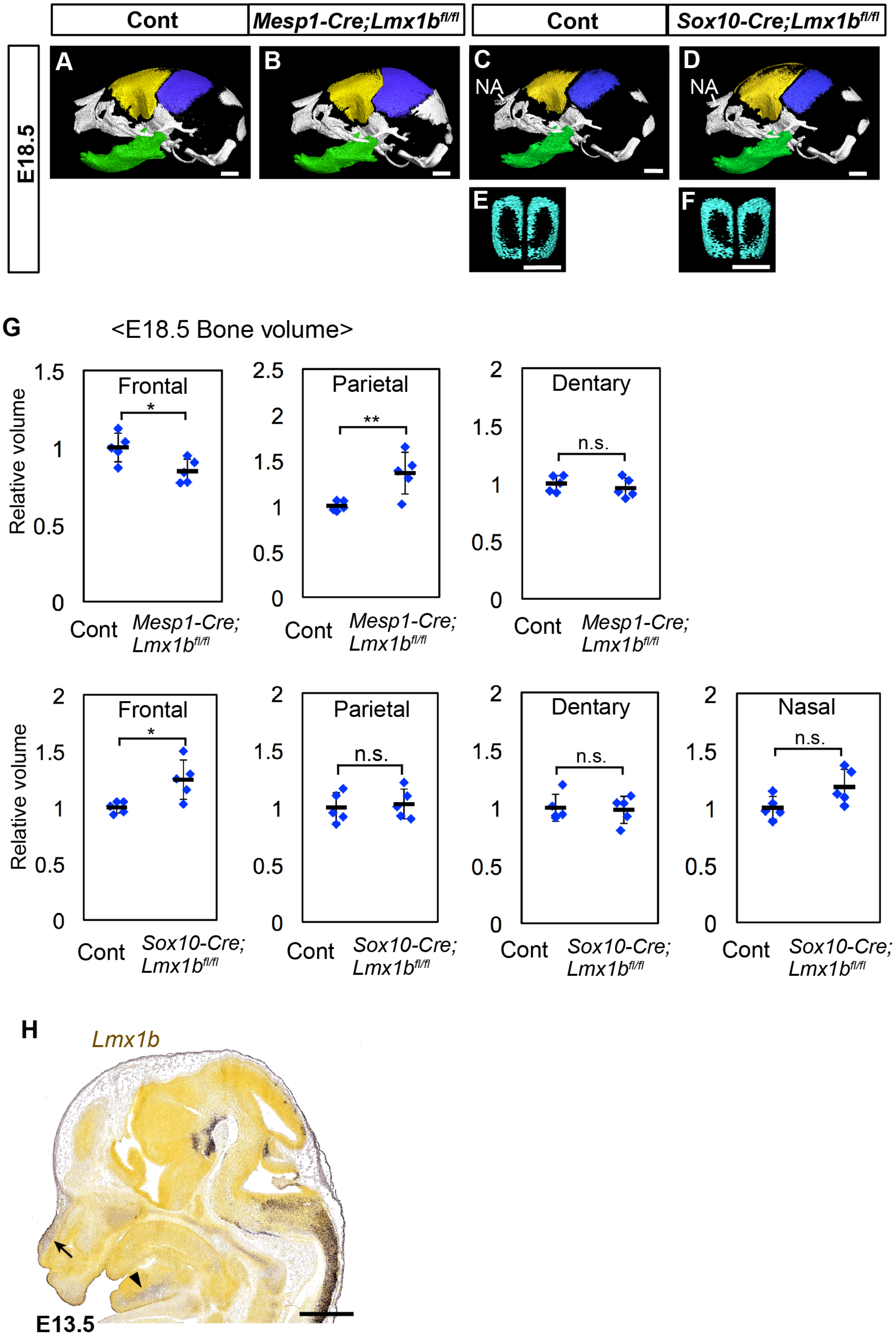

Supplementary Figure 2. Calvarial phenotype of mesoderm-specific and neural crest-specific *Lmx1b* deletion mutants - additional data

(A-F) 3D reconstruction from microCT scans of E18.5 skulls. A-D are the same as Figure 1A,B,K,L. E and F are superior views of the nasal bone (NA in C,D). Yellow: frontal bone, blue: parietal bone, green: dentary bone. (G) Volumes of each piece of bone measured from microCT data, and normalized to an average volume from control samples (= 1). (H) From Allen Mouse Brain Atlas (Allen Institute for Brain Science 2004), specimen 320-3462. A sagittal section of an E13.5 wild type embryo head processed by RNA in situ hybridization for *Lmx1b*. The arrow and the arrowhead in H point to *Lmx1*b expression in the nasal and the mandibular mesenchyme, respectively, Bar: 1 mm. *: $p<0.05$, **: $p<0.01$, n.s.: not statistically significant ($p>0.05$).

Figure S3

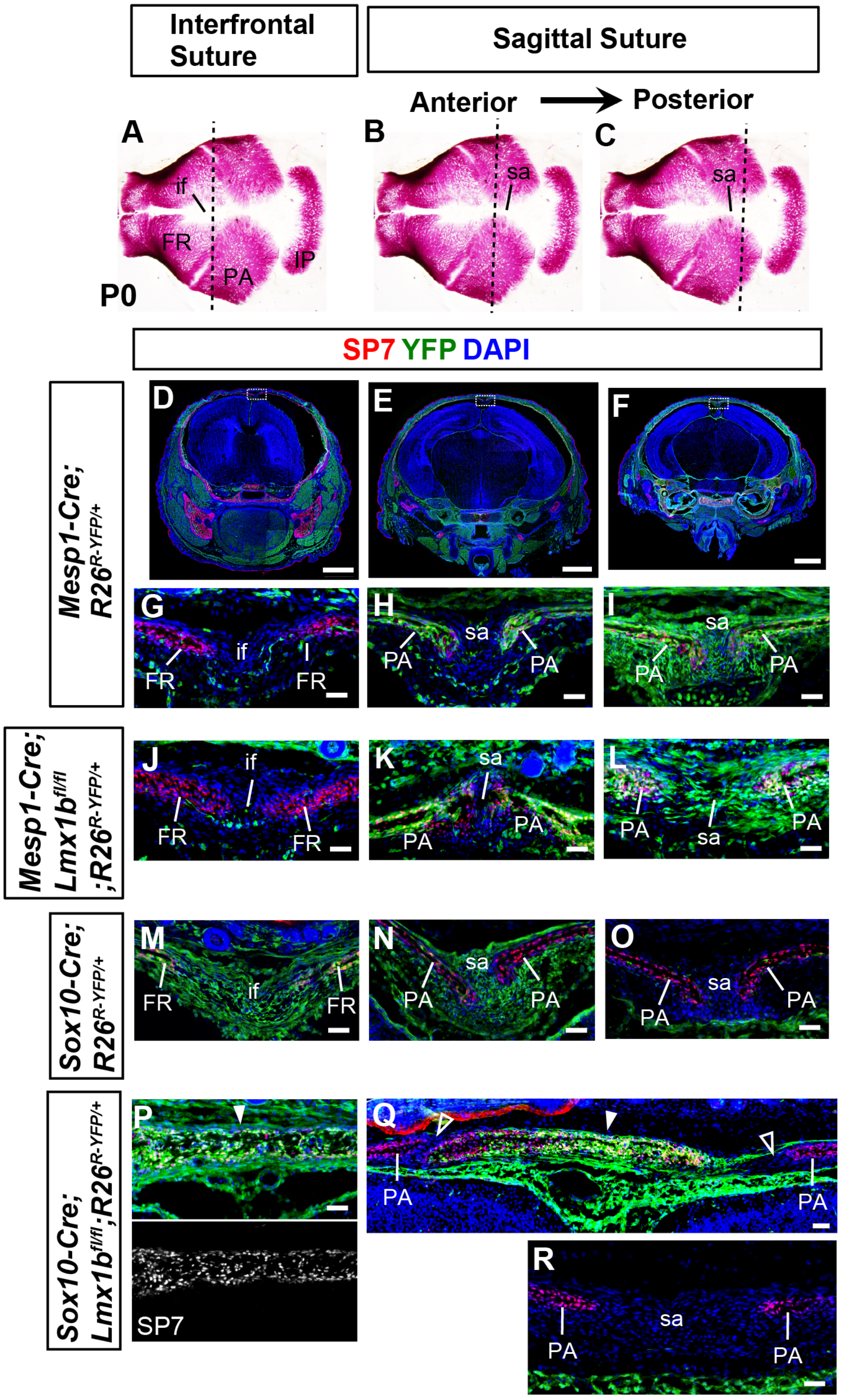

Supplementary Figure 3. Contribution of mesoderm-derived and neural crest-derived cells to the interfrontal suture and the sagittal suture

(A-C) Normal skulls at P0 stained with Alizarin red for bone. (D-R) Coronal sections of P0 heads processed by immunofluorescence for SP7 and YFP. The dotted lines in A-C indicate an approximate plane of section, in an anterior-to-posterior progression, for all the panels in each column in D-P. Q

and R are from the section positions indicated in B and C, respectively. The boxes in D-F mark the tissue regions shown in G-I, respectively, and the panels below in the same column in J-P are from equivalent regions of the animals of the indicated genotype. Q and R are from regions equivalent to N and O, respectively. 3 samples per genotype were examined for each plane of section. The arrowheads in P and Q point to the heterotopic bone at the midline. The open arrowheads in Q point to the suture mesenchyme between the midline bone and the parietal bone. Bar in D-F: 1 mm, bar in G-R: 0.05 mm. FR: frontal bone, if: interfrontal suture, IP: interparietal bone, PA: parietal bone, sa: sagittal suture.

Figure S4

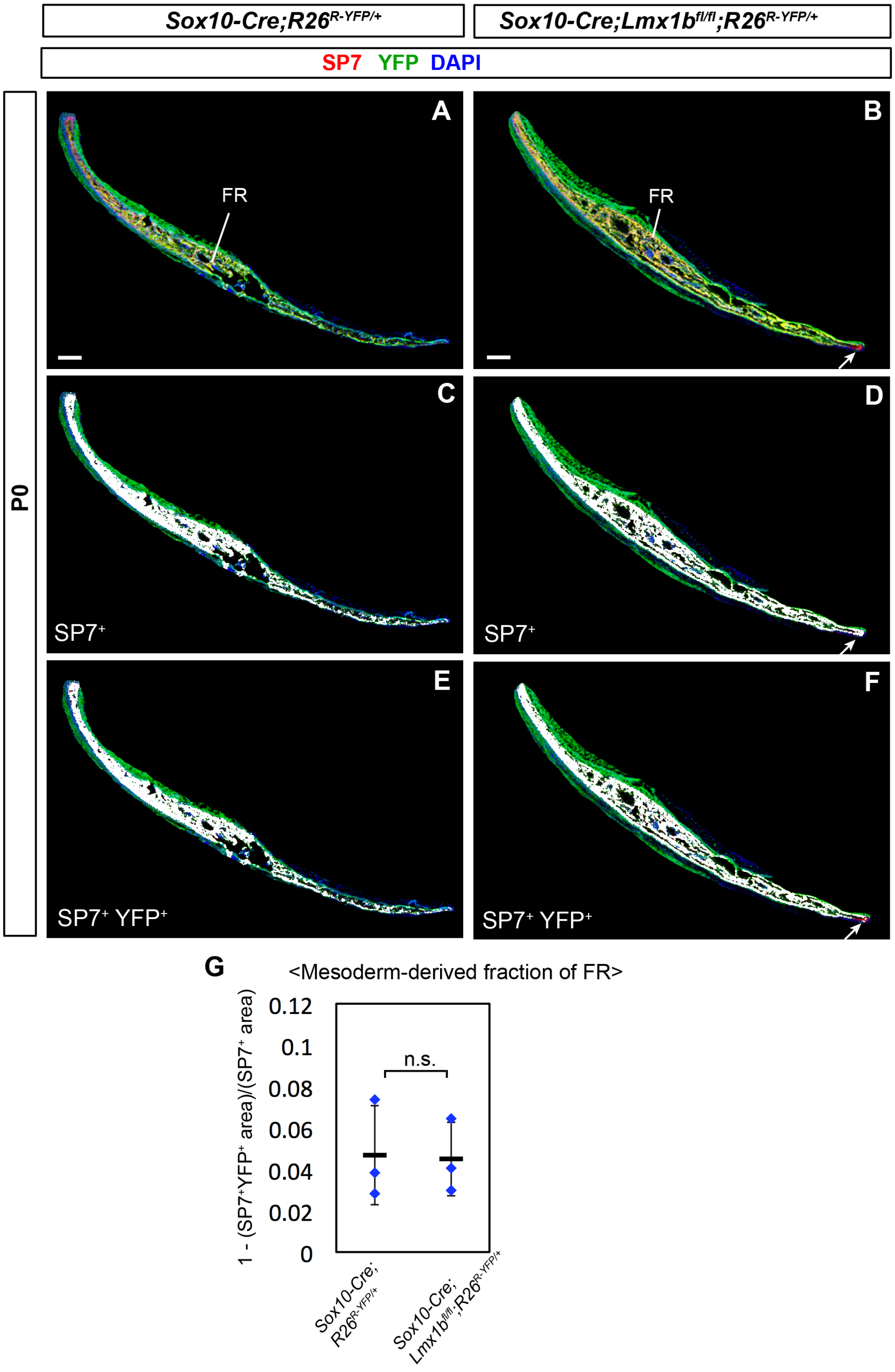

Supplementary Figure 4. Measuring the lineage composition of the frontal bone.

(A,B) Frontal bone areas from transverse sections of P0 heads processed by immunofluorescence for SP7 and YFP. The sections are from a position equivalent to the one in Fig. 2F. (C,D) $SP7^+$ areas selected at FIJI (white) using a threshold for the pixel intensity of the red channel. (E,F) $SP7^+YFP^+$ area selected at FIJI (white) using a threshold for the pixel intensity of both red and green channels. Bar: 0.2 mm. The arrows in B,D,F point to the mesoderm-derived (YFP^-) cells at the posterior tip of the frontal bone. (G) Comparison of mesoderm-derive fraction of the frontal bone, calculated by subtracting the neural crest-derived fraction from 1. 3 samples per genotype were examined. n.s.: not significantly different ($p>0.05$).

Figure S5

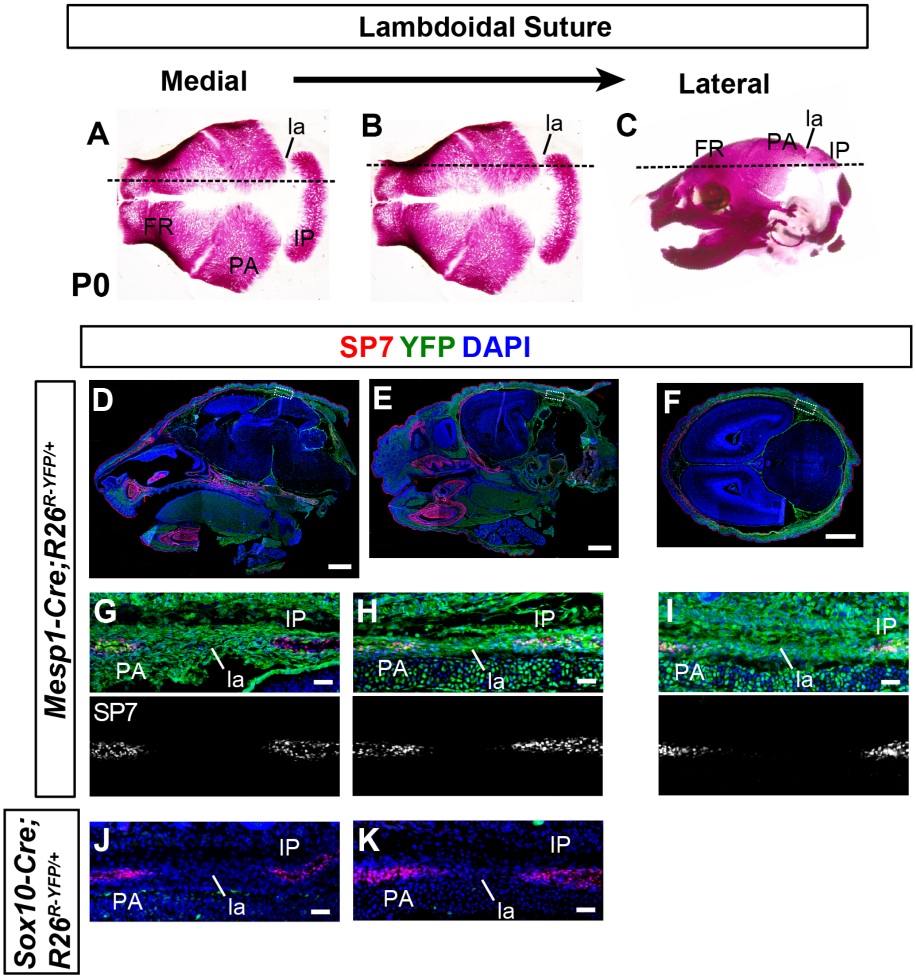

Supplementary Figure 5. Contribution of mesoderm-derived and neural crest-derived cells to the lambdoidal suture

(A-C) Normal skulls at P0 stained with Alizarin red for bone. The dotted lines in A-C indicate an approximate plane of section for all the panels in each column, cutting through the medial part of the lambdoidal suture (la) in A and progressing laterally. (D-F) Coronal sections (D,E) and a transverse section (F) of P0 *Mesp1-Cre;R26*$^{R\text{-}YFP/+}$ heads processed by immunofluorescence for SP7 and YFP. The boxes in D-F mark the tissue regions shown in G-I, respectively, and the panels below in the same column (J,K) are from equivalent regions of the animals of the indicated genotype. 3 samples per genotype were examined for each plane of section. In *Sox10-Cre;R26*$^{R\text{-}YFP/+}$ animals, we did not detect YFP^{+} cells in the interparietal bone (IP) either in the medial (J) nor in the lateral part (K), which indicates that *Sox10-Cre* is not active in all neural crest-derived cells in this part of the calvaria. Bar in D-F: 1 mm, bar in G-K: 0.05 mm.

Figure S6

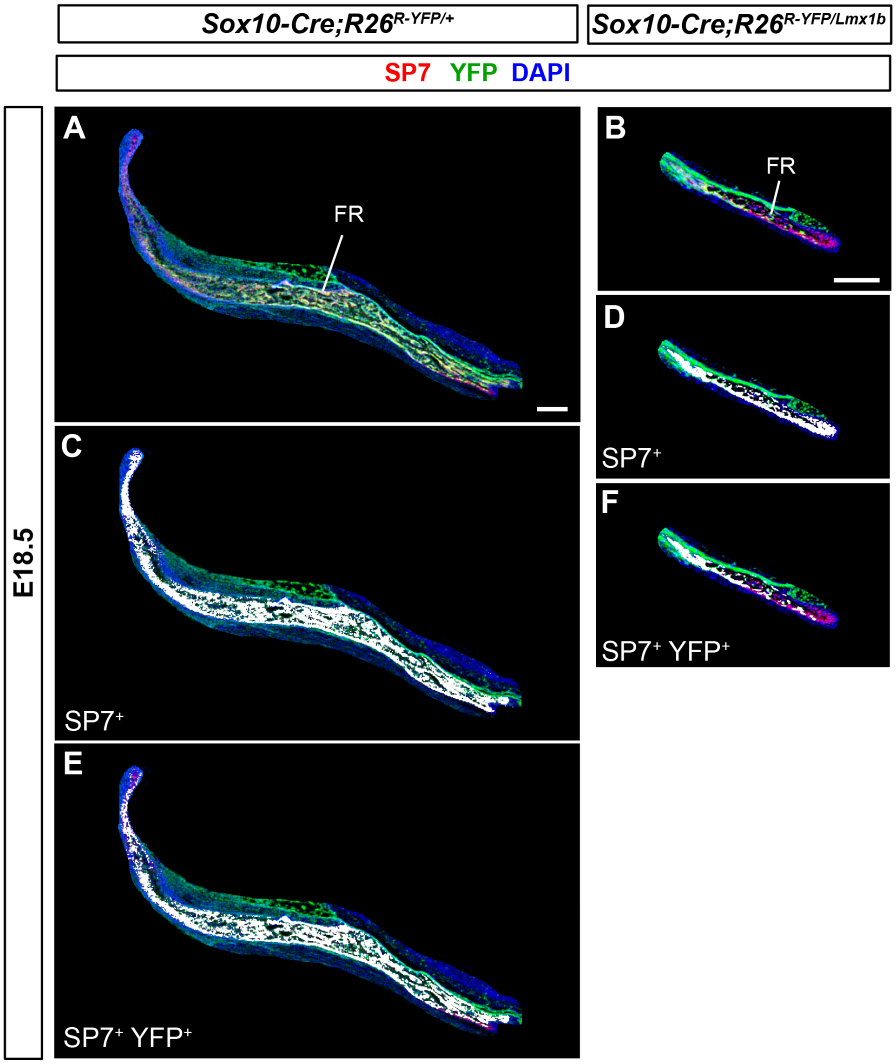

Supplementary Figure 6. Measuring neural crest-derived fraction of the frontal bone.

(A,B) Frontal bone areas from Figure 6C and 6D, respectively. (C,D) $SP7^+$ areas selected at FIJI (white) using a threshold for the pixel intensity of the red channel. (E,F) $SP7^+YFP^+$ area selected at FIJI (white) using a threshold for the pixel intensity of both red and green channels. Bar: 0.2 mm.

Figure S7

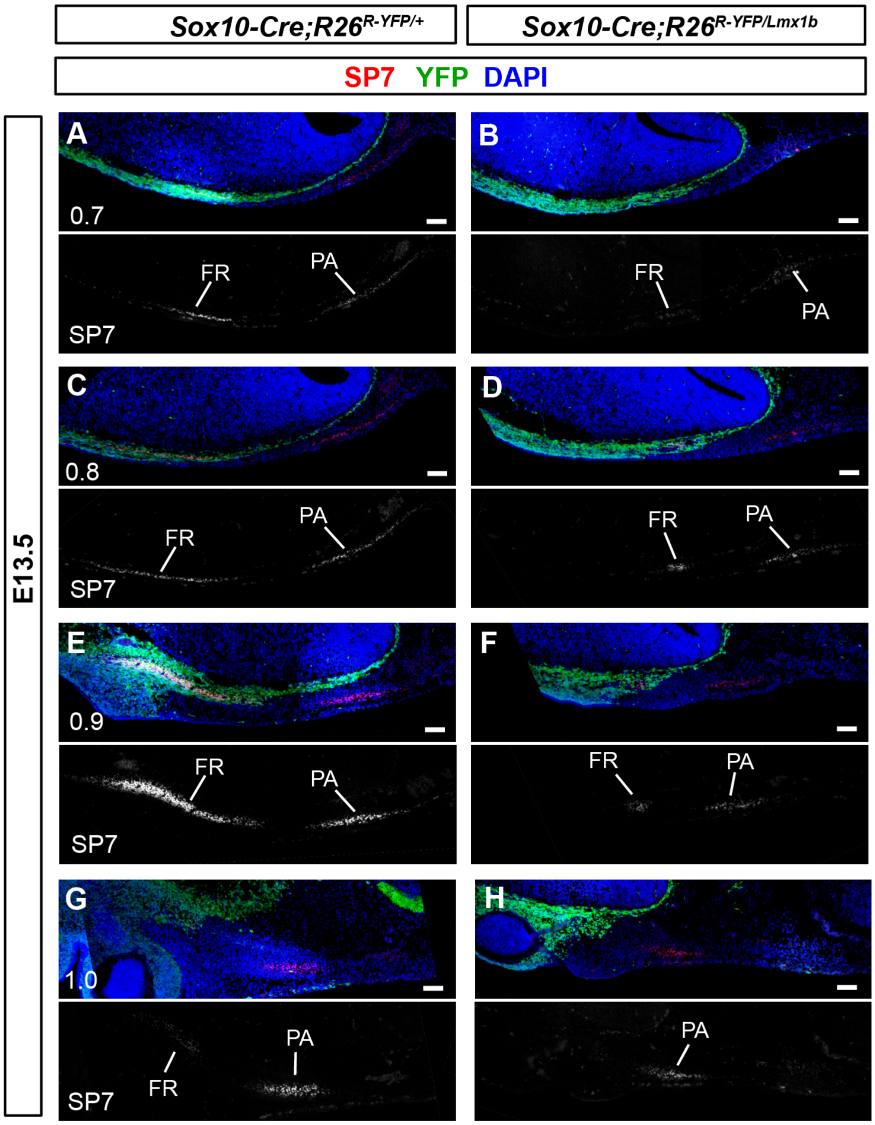

Supplementary Figure 7. Defects in osteogenesis in neural crest-specific *Lmx1b* overexpression mutants at E13.5

(A-H) Apical-basal series of sections from the embryos shown in Fig. 6K and L, processed by immunofluorescence for SP7 and YFP. The numbers in the bottom-left corner of each panel indicate the position of the sections as defined in Figure 4A. Bar: 0.1 mm.
